# Group 2 innate lymphoid cells protect mouse heart from myocardial infarction injury via interleukin 5, eosinophils, and dendritic cells

**DOI:** 10.1093/cvr/cvac144

**Published:** 2022-09-05

**Authors:** Tianxiao Liu, Zhaojie Meng, Jing Liu, Jie Li, Yuanyuan Zhang, Zhiyong Deng, Songyuan Luo, Minjie Wang, Qin Huang, Shuya Zhang, Pauline Fendt, Julie Devouassoux, Dazhu Li, Andrew Neil James McKenzie, Matthias Nahrendorf, Peter Libby, Junli Guo, Guo-Ping Shi

**Affiliations:** Guangdong Provincial Geriatrics Institute, Guangdong Provincial People’s Hospital, Guangdong Academy of Medical Sciences, Guangzhou 510080, China; Department of Medicine, Brigham and Women’s Hospital and Harvard Medical School, Boston, MA 02115, USA; Laboratory of Cardiovascular Immunology, Institute of Cardiology, Union Hospital, Tongji Medical College, Huazhong University of Science and Technology, Wuhan 430022, China; Department of Medicine, Brigham and Women’s Hospital and Harvard Medical School, Boston, MA 02115, USA; Department of Medicine, Brigham and Women’s Hospital and Harvard Medical School, Boston, MA 02115, USA; Laboratory of Cardiovascular Immunology, Institute of Cardiology, Union Hospital, Tongji Medical College, Huazhong University of Science and Technology, Wuhan 430022, China; Department of Medicine, Brigham and Women’s Hospital and Harvard Medical School, Boston, MA 02115, USA; Department of Medicine, Brigham and Women’s Hospital and Harvard Medical School, Boston, MA 02115, USA; Institute of Cardiovascular Research, Key Laboratory of Emergency and Trauma of Ministry of Education, Hainan Medical University, Haikou 570100, China; Department of Medicine, Brigham and Women’s Hospital and Harvard Medical School, Boston, MA 02115, USA; Department of Medicine, Brigham and Women’s Hospital and Harvard Medical School, Boston, MA 02115, USA; Department of Medicine, Brigham and Women’s Hospital and Harvard Medical School, Boston, MA 02115, USA; Department of Medicine, Brigham and Women’s Hospital and Harvard Medical School, Boston, MA 02115, USA; Department of Medicine, Brigham and Women’s Hospital and Harvard Medical School, Boston, MA 02115, USA; Institute of Cardiovascular Research, Key Laboratory of Emergency and Trauma of Ministry of Education, Hainan Medical University, Haikou 570100, China; Department of Medicine, Brigham and Women’s Hospital and Harvard Medical School, Boston, MA 02115, USA; Department of Medicine, Brigham and Women’s Hospital and Harvard Medical School, Boston, MA 02115, USA; Laboratory of Cardiovascular Immunology, Institute of Cardiology, Union Hospital, Tongji Medical College, Huazhong University of Science and Technology, Wuhan 430022, China; Medical Research Council Laboratory of Molecular Biology, Cambridge, UK; Center for Systems Biology, Massachusetts General Hospital, Harvard Medical School, Boston, MA 02114, USA; Department of Medicine, Brigham and Women’s Hospital and Harvard Medical School, Boston, MA 02115, USA; Department of Medicine, Brigham and Women’s Hospital and Harvard Medical School, Boston, MA 02115, USA; Institute of Cardiovascular Research, Key Laboratory of Emergency and Trauma of Ministry of Education, Hainan Medical University, Haikou 570100, China; Department of Medicine, Brigham and Women’s Hospital and Harvard Medical School, Boston, MA 02115, USA

**Keywords:** ILC2, myocardial infarction, interleukin 5, eosinophil, dendritic cell

## Abstract

**Aims:**

Group 2 innate lymphoid cells (ILC2s) regulate adaptive and innate immunities. In mouse heart, production of myocardial infarction (MI) increased ILC2 accumulation, suggesting a role for ILC2 in cardiac dysfunction post-MI.

**Methods and results:**

We produced MI in ILC2-deficeint *Rora^fl/fl^Il7r^Cre/+^* mice and in *Icos^fl-DTR-fl/+^Cd4^Cre/+^* mice that allowed diphtheria toxin-induced ILC2 depletion. Genetic or induced deficiency of ILC2 in mice exacerbated cardiac dysfunction post-MI injury along with increased myocardial accumulation of neutrophils, CD11b^+^Ly6C^hi^ monocytes, and CD4^+^ T cells but deficiency of eosinophils (EOS) and dendritic cells (DC). Post-MI hearts from genetic and induced ILC2-deficient mice contained many more apoptotic cells than those of control mice, and *Rora^fl/fl^Il7r^Cre/+^* mice showed thinner and larger infarcts and more collagen-I depositions than the *Il7r^Cre/+^* mice only at early time points post-MI. Mechanistic studies revealed elevated blood IL5 in *Il7r^Cre/+^* mice at 1, 7, and 28 days post-MI. Such increase was blunted in *Rora^fl/fl^Il7r^Cre/+^* mice. Administration of recombinant IL5 reversed EOS losses in *Rora^fl/fl^Il7r^Cre/+^* mice, but IL5 did not correct the DC loss in these mice. Adoptive transfer of ILC2, EOS, or DC from wild-type mice, but not ILC2 from *Il5^−/−^* mice improved post-MI cardiac functions in *Rora^fl/fl^Il7r^Cre/+^* recipient mice. EOS are known to protect cardiomyocytes from apoptosis. Here we showed that DC acted like EOS in blocking cardiomyocyte apoptosis. Yet, ILC2 or IL5 alone did not directly affect cardiomyocyte apoptosis or TGF-β (transforming growth factor-β)-induced cardiac fibroblast Smad signalling.

**Conclusion:**

This study revealed an indirect cardiac reparative role of ILC2 in post-MI hearts via the IL5, EOS, and DC mechanism.

## Graphical Abstract

**Graphical abstract:**
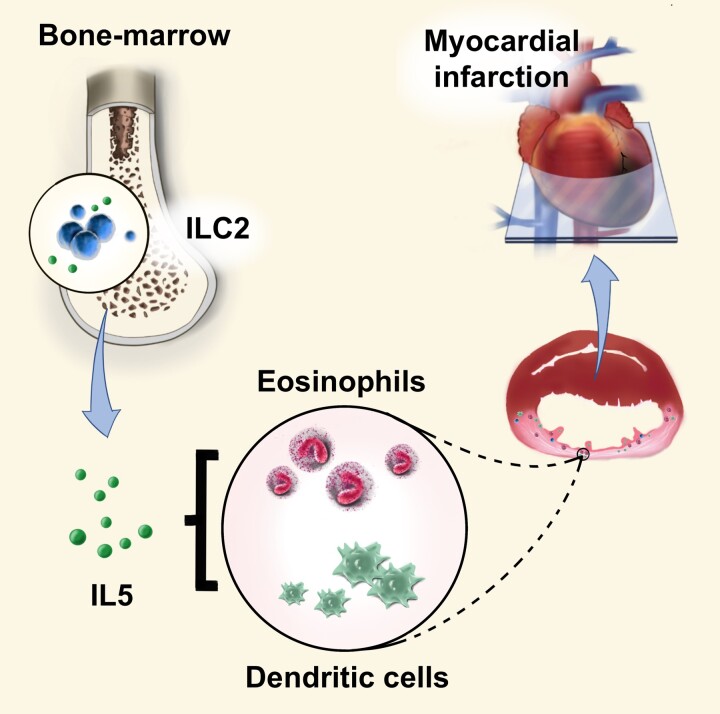

## 1. Introduction

Myocardial infarction (MI) is a major cause of morbidity and mortality worldwide and continues to pose significant therapeutic challenges.^1^ Accumulating evidence suggests that MI is an inflammatory disease.^2^ Inflammatory cell recruitment to the heart plays a pivotal role in post-MI cardiac remodeling and heart failure. When MI occurs, necrotic cardiomyocytes release danger signals, which activate innate immune pathways and trigger an intense inflammatory response. These immune cells release both pro- and anti-inflammatory mediators. Not only the degree of immune cell recruitment to the heart but also the balance between different types or subtypes of cells contributes to cardiac remodeling post-MI. The suppression of pro-inflammatory response and reinforcement of the anti-inflammatory repair are associated with improved prognosis of MI.^2,3^ Therapeutic modulation of the inflammatory and reparative responses may hold promise for prevention of post-infarction heart failure.

Group 2 innate lymphoid cells (ILC2) are a newly identified subset of immune cells that produce immune effector cytokines to regulate adaptive immunity.^4,5^ ILC2s are defined by lack of expression of myeloid or lymphoid lineage markers such as CD11b, CD11c, Gr1, CD3, etc., but expression of inducible T-cell costimulator (ICOS), ST2 (suppression of tumorigenicity 2, interleukin-33 receptor), CD127 (interleukin 7 receptor, IL7R), killer cell lectin-like receptor G-1 (KLRG1), CD25 (IL2 receptor), IL17BR (IL25 receptor), and CD90 (Thy1). ILC2s are innate lymphocytes that develop in the bone-marrow under the control of the transcription factor GATA-3 and the nuclear receptor RAR-related orphan receptor-α (RORα).^5^ Most ILC2 are found in the epithelial barrier tissues including skin, gut, and lungs, although they also present in adipose tissues, joints, and other sites.^6^ Injury or inflammation activates epithelial cells to produce IL33 and IL25 and thymic stromal lymphopoietin (TSLP), which then promote ILC2 to produce type-2 cytokines IL4, IL5, and IL13.^7^ Limited information is available regarding ILC2 function in cardiovascular diseases (CVDs). Indirect evidence shows that IL2-anti-IL2 complexes, IL33, IL25, and TSLP increase ILC2 content in aortic lesions, spleen, and plasma ILC2 cytokines (IL5, IL13) and reduce atherosclerosis in *Ldlr^−/−^* or *Apoe^−/−^* mice.^8–11^*Ldlr^−/−^* mice received bone-marrow transplantation (BMT) from *Il13^−/−^* mice developed much larger atherosclerotic lesions than those received BMT from wild-type (WT) mice.^12^ In mice fed a high fat diet, ILC2 were found in the peri-aortic adventitial adipose tissue where they expressed high levels of IL5 and IL13.^13^ Those ILC2s were Lin^−^ICOS^+^CD25^+^ CD127^+^KLRG1^+^ST2^+^CD90^+^Sca-1^+^cKit^+^ and produced IL5 to trigger B1a cell proliferation and natural IgM (immunoglobulin M) production.^13,14^ Yet, anti-CD90.2-mediated ILC2 depletion did not affect plaque progression.^8^ It was proposed that anti-CD90.2 antibody only depleted ILC2 in the lymphoid organs, but not those in other tissues.^15^ Nuclear receptor RORα is required for ILC2 development. ILC2-deficient mice were described where RORα is deleted in cells expressing IL7R (*Rora^fl/fl^Il7r^Cre/+^*).^16^*Ldlr^−/−^* mice received BMT from *Rora^fl/fl^Il7r^Cre/+^* mice showed reduced ILC2 in bone-marrow and mesenteric lymph node, and developed larger atherosclerotic lesions with more lesion CD3^+^ T cells and foam cells than those received BMT from WT mice.^17^ Yet, it remains unknown whether reduced atherosclerosis in mice received BMT from ILC2-deficient mice was due to ILC2 deficiency or changes of other immune cells because of ILC2 deficiency. Further, the mechanism by which BMT from ILC2-deficient mice contributed to atherogenesis in *Ldlr^−/−^* mice remains untested.

In this study, we revealed ILC2 accumulation in mouse hearts post-MI. We used the ILC2-deficient *Rora^fl/fl^Il7r^Cre/+^* mice and diphtheria toxin (DTX)-induced ILC2-depleted *Icos^fl−DTR−fl/+^Cd4^Cre/+^* (ICOS-T) mice to test whether absence of ILC2 affects post-MI cardiac function. We also examined whether ILC2-derived IL5 and its downstream cells, such as eosinophils (EOS) and dendritic cells (DCs) are part of the mechanisms of ILC2 functions in post-MI cardiac repair.

## 2. Methods

The data, analytic methods, and study materials that support the findings of this study are available from the corresponding authors upon reasonable request.

### 2.1 Mice

C57BL/6 WT mice (000664), *Il5^−/−^* mice (003175), and 45.1 transgenic mice (002014) aged 7–8 weeks were purchased from the Jackson laboratory (Bar Harbor, ME). The Group 2 innate lymphoid cells (ILC2)-deficient mice *Rora^fl/fl^Il7r^Cre/+^*(carrying a Rora deletion in Il7ra-expressing lymphocytes that leads to ILC2 deficiency in the absence of neurological defects) and ICOS-T (carrying a loxP-flanked diphtheria toxin receptor (DTR) gene inserted into the ICOS locus, which is expressed ‘preferentially’ on T cells and ILC2 cells, that enables *CD4^Cre/+^*–mediated excision of the DTR gene from T cells and its retention in ILC2 cells, enabling ILC2 depletion through DTX and sparing of T cells) were previously reported.^16^ Mice were used for experiments at age 8–10 weeks old. Mice were anesthetized with isoflurane inhalation and received one dose of meloxicam by subcutaneous injection (3 mg/kg) before surgery. After surgery, mice received subcutaneous meloxicam (3 mg/kg) at one dose each 24 h for 48 h and subcutaneous buprenorphine (0.05 mg/kg) at one dose per 12 h for 48 h. All animal procedures conformed to the Guide for the Care and Use of Laboratory Animals published by the US National Institutes of Health and was approved by the Brigham and Women’s Hospital Standing Committee on Animals (protocol #2016N000442).

### 2.2 MI

Mouse MI surgery was performed as previously described.^18^ Briefly, mouse was anaesthetized with 1.5% isoflurane, orally intubated, and then connected to a rodent ventilator. After removing the fur and opening the left thorax, the heart was exposed and MI was produced by permanent ligation of the left anterior descending (LAD) artery using a 7-0 silk suture (Ethicon, Somerville, NJ). Bleaching of the distal myocardium verified ischemia. Sham-operated mice underwent the same procedure without LAD ligation. Mice within 1 to 10 days of operation were used to analyze heart immune cell infiltration. Mice at 1, 7, and 28 days post-MI 30 days were used to analyze the heart function with a pre-harvest echocardiogram being performed for reference. Hearts were used for real-time PCR, immunoblot, and immunohistological or immunofluorescent staining.

### 2.3 Treatment and groups

To determine the infiltration of ILC2 in infarcted hearts, male WT mice (C57BL/6) aged 8–10 weeks were subject to MI or sham operation. Mouse heart and spleen were harvested at different days (1, 3, 7, 10) post-MI for FACS analysis. To assess the role of ILC2 in MI, ILC2-deficient male mice *Rora^fl/fl^Il7r^Cre/+^* and control male mice *Il7r^Cre/+^* aged 8–10 weeks were used for MI and sham operation. Mice were harvested at 1, 7, or 28 days post-MI. Before harvest, echocardiogram was performed to test cardiac function. Body weight, heart weight, tibia length were measured at harvest. The heart was collected to prepare frozen sections for histological staining, including haematoxylin and eosin (H&E) and Sirius red staining. The spleen was collected for immune cell analysis. Plasma was collected and stored for ELISA (enzyme-linked immunosorbent assay) testing. To test the role of ILC2-derived IL5, EOS, and DC in mouse MI, we performed adoptive transfer of WT ILC2 (2 × 10^5^/mouse), *Il5^−/−^* ILC2 (2 × 10^5^/mouse), WT EOS (1 × 10^7^/mouse), and WT DC (1 × 10^6^/mouse) respectively in 200 µl phosphate buffered saline (PBS) to *Rora^fl/fl^ Il7r^Cre/+^* recipient mice by tail intravenous (i.v.) injection at 30 min before MI surgery. Control mice received the same volume of PBS. To induce ILC2 depletion in ICOS-T (*Icos^+/−^Cd4^Cre/+^*) mice, mice were intraperitoneally injected with DTX (0564, Sigma-Aldrich, St. Lois, MO) at a concentration of 300 ng in 200 µl PBS per mouse per day for 3 days to induce ILC2 depletion before the surgeries. DTX was given twice a week for the first 2 weeks post-MI. The control *CD4^Cre/+^* mice or ICOS-T mice received the same volume of vehicle underwent the same surgeries. Hearts from *Rora^fl/fl^Il7r^Cre/+^*, *Il7r^Cre/+^*, ICOS-T that received DTX or vehicle were also collected at 1 day post-MI to assess cardiac cell apoptosis.

### 2.4 Echocardiogram

A Vevo 3100 high-frequency micro-ultrasound imaging system with a 70-MHz transducer (VisualSonics, Toronto, Canada) was used to measure mouse cardiac function. Mice were conscious and properly positioned and restrained on a board during the examination. Two-dimensional echocardiographic views of the mid-ventricular short axis and parasternal long axes M-mode was obtained at baseline and at 1, 7, and 28 days post-MI. M-mode tracings at mid-papillary muscle level were recorded to measure left ventricle (LV) wall thickness, LV end-diastolic diameter (LV EDD), and LV end-systolic diameter (LV ESD). Percentage of fraction shortening (%FS) and percentage of ejection fraction (%EF) and other cardiac functions were calculated by the built-in software package.

### 2.5 Heart immune cell and splenocyte preparations and flow cytometry

Mouse heart was perfused with 20 mL of cold PBS and carefully removed, minced into small pieces and digested in a 0.1% collagenase B solution (LS004177, Worthington Biochemical Co, Lakewood, NJ) for 30 min in a 37°C water bath. Digestion was vortexed every 10 min. After digestion, single cells were neutralized by RPMI 1640 medium supplemented with 10% fetal bovine serum (FBS) and filtered through a 70-µm cell strainer. Heart immune cells were separated using density gradient centrifugation in a 15-mL tube layered with 2 mL 100% Percoll (17-0891-09, Fisher Scientific, Hampton, NH), 1.5 ml 80% Percoll, 1.5 mL 62% Percoll, 1.5 mL 55% Percoll, and 3 mL 45% Percoll that was pre-mixed with the cell preparation. Cells were centrifuged at 800 g for 30 min. Single cells between each layer were carefully obtained and washed with PBS twice. Splenocytes were prepared by mincing the spleen in a petri dish in 5∼10 ml of PBS, filtered through a 70-µm cell strainer, precipitated, and lysed in a red cell lysis buffer (A10492-01, Fisher Scientific). Cells were washed with PBS. To measure ILC2 in heart tissue or spleen, heart single cells or splenocytes were stained with cell viability dye (65-0866-14), CD45 (25-0451-82), mouse hematopoietic lineage antibody cocktail (LIN, 88-7772-72), ICOS (25-9942-82), KLRG1 (46-5893-82) and CD127 (17-1271-82). To test neutrophil, Ly6C^hi^ and Ly6C^lo^ monocytes, DC, CD4^+^ and CD8^+^ T cells, heart cells or splenocytes were stained with cell viability dye (65-0866-14 and 65-0863-14) and cell surface marker antibodies including CD45 (25-0451-82 and 11-0451-85), CD45.1 (45-0453-82), CD11b (17-0112-82), Siglec-F (12-1702-82), Gr-1 (12-5931-82), Ly6C (53-5932-82), CD11c (53-0114-82), MHC-II (107607, BioLegend, San Diego, CA), CD4 (A15384), and CD8 (12-0081-82) (all from eBioscience, San Diego, CA). In each experiment, the appropriate isotype control monoclonal antibodies and single conjugate controls were also included.

### 2.6 Mast cell toluidine blue staining

Mouse heart frozen sections were stained with 3% toluidine blue in PBS for 2 min. Sections were rinsed in PBS few times until purple color mast cells appeared. Slides were covered with coverslip, images were collected, and mast cells were counted under the microscope.

### 2.7 Mouse ILC2 isolation and cell sorting

Mouse ILC2 were isolated and sorted as previously described.^19,20^ In brief, mice aged 8–10 weeks were first intraperitoneally injected with IL33 at a concentration of 500 ng/mouse/day for 3 days. Mesenteric lymph nodes (MLNs) and spleen were then carefully removed after perfusing mice with 20 ml of cold PBS. MLN and spleen were grinded into single cells followed by filtering through a 70-µm cell strainer. Single cell suspension was then separated by density gradient centrifugation in a 15-mL tube layered with 5 ml lymphocyte separation medium (ICN50494, MP Biomedicals, Irvine, CA) and 5 ml cells suspension pre-mixed with PBS. After centrifugation at 400 g for 30 min, the lymphocyte layer between lymphocyte separation medium and PBS was carefully collected and washed with PBS and autoMACS running buffer (130-091-221, Miltenyi Biotec Inc. Cambridge, MA) twice, respectively. Innate lymphoid cells were then enriched using the lineage cell depletion kit (130-090-858, Miltenyi Biotec Inc.) according to the manufacturer’s protocol. The enriched cells were then stained with cell viability dye (65-0866-14), CD45 (25-0451-82), mouse hematopoietic lineage antibody cocktail (LIN, 88-7772-72), ICOS (25-9942-82), KLRG1 (46-5893-82) and CD127 (17-1271-82) at 4°C for 30 min. Antibodies were all from Thermo Fisher scientific (Waltham, MA). ILC2 were sorted using a FACSAria-SORP cell sorter. To prepare the ILC2 lysate for cell treatment, 2 × 10^5^ ILC2 were resuspended in 1 mL of culture medium. After 5 cycles of freezing and thawing, cells were centrifuged at 3000 rpm. Supernatants were then collected and stored at −80°C.

### 2.8 Mouse bone-marrow-derived EOS and DC culture

Mouse EOS were cultured as previously reported.^21^ In brief, bone-marrow cells were collected from the femurs and tibias of WT C57BL/6 mice, followed by centrifuging for 5 min at 300 g. Red blood cells were lysed by pipetting cell pellet up and down twice in 9 mL ddH_2_O followed by the addition of 1 ml 10xPBS. The lysis protocol was repeated for a maximum 3 times. After centrifugation, the bone-marrow cells were suspended in 10 ml PBS containing 0.1% BSA and filtered through a 70-µm cell strainer after which cell counting was performed. The bone-marrow cells were cultured at a concentration of 10^6^/ml in a base medium containing RPMI 1640 (61870036, Gibco, Thermo Fisher scientific) supplemented with 20% FBS (10082147, Gibco), 100 IU/ml penicillin and 10 μg/ml streptomycin (15140122, Gibco), 2 mM glutamine (25030081, Gibco), 25 mM HEPES (15630080, Gibco), 1x nonessential amino acids (11140050, Gibco), 1 mM sodium pyruvate (11360070, Gibco), and 50 μM 2-mercaptoethanol (516732, Sigma-Aldrich, Louis, MO). From days 0 to 4, cells were supplemented with 100 ng/ml mouse stem cell factor (SCF, 100 ng/ml, 250-03, PeproTech, Rocky Hill, NJ) and mouse Flt-3-ligand (100 ng/ml, 250-31L, PeproTech). On day 2, one-half of the media from each flask was replaced with fresh medium containing SCF and Flt-3-ligand. On day 4, the medium containing SCF and FLT3-L was replaced with base medium containing 10 ng/ml recombinant mouse IL5 (405-ML, R&D Systems, Minneapolis, MN). On day 8, the cells were moved to new flasks and maintained in fresh medium supplemented with IL5. Half of the culture media was changed with fresh media containing IL5 every two days until day 14. On day 14, cells were collected and cell purity was examined by FACS. For the differentiation of DC, bone-marrow cells were supplemented with recombinant murine granulocyte-macrophage colony-stimulating factor (GM-CSF, 20 ng/ml, 315-03, PeproTech) and IL4 (100 ng/ml, 214-14, PeproTech). The medium was changed every three days. At day 12, the mature DC were collected for further studies.^22,23^ To prepare the EOS or DC lysate for cell treatment, 1 × 10^7^ EOS or DC were resuspended in 1 mL culture medium. After 5 cycles of freezing and thawing, cells were centrifuged at 3000 rpm. Supernatants were then collected and stored at −80°C.

### 2.9 Adult mouse cardiomyocyte and cardiac fibroblast isolation

Adult mouse cardiomyocytes were isolated and cultured as previously reported.^18,24^ In brief, mouse (C57BL/6 background) was pre-treated with intraperitoneal injection of 100 µl heparin (63323054207, APP Pharmaceuticals, LLC, Schaumburg, IL) for 5–8 min. After the mouse was fully euthanized with isoflurane, the heart with aorta was carefully removed and immediately placed into a dish containing perfusion buffer containing 22% NaCl, 3.5% KCl, 0.82%KH_2_PO_4_, 0.85% Na_2_HPO_4_, 3% MgSO4, 0.12% phenol red, 31.56% NaHCO3, 10.1% KHCO3, 1 M HEPES, 0.375% taurine, 500 mM 2,3-butanedione monoxime (BDM) and 0.1% glucose at room temperature. The extraneous tissues around the aorta were carefully and quickly removed and dissected. The heart was then cannulated quickly and perfused with the perfusion buffer in a perfusion system as described.^18,24^ After the perfusion buffer becomes clear, the perfusion buffer was replaced by digestion buffer which was pre-made with the perfusion buffer supplemented with 0.1% collagenase B (LS004177, Worthington Biochemical Co). Digestion was performed about 10 min until the heart became pale, swollen, and flaccid. Once the digestion was complete, myocyte dissociation was performed in the hood and followed with calcium reintroduction as we described previously.^18,24^ Cardiomyocytes were then planted at 1 × 10^5^ cells per well in planting medium containing 10% FBS, 10 mM BDM and 1% ATP on a laminin (1–2 μg/mL)-pre-coated 6-well plate and incubated in a 2% CO_2_ incubator at 37°C for 2 h to make the attachment about 80%. After attachment, plating medium was gently removed and replaced with culture medium containing 1% BSA and 10 mM BDM. All medium preparations were done as described.^18,24^ Adult mouse cardiomyocytes were pre-treated with different concentrations of ILC2 lysate (equivalent to 0, 10^3^, 10^4^, 5 × 10^4^ ILC2/mL), EOS lysate (equivalent to 1 × 10^6^ EOS/mL), DC lysate (equivalent to 2 × 10^5^ DC/mL) from WT mice, recombinant IL5 (10 ng/mL), or different combinations. Cardiomyocytes were then treated with 100 µM H_2_O_2_ or sterile water for 4 h to induce apoptosis. Cell apoptosis was detected by immunoblot analysis to detect cleaved caspase-3 or Bcl-2 or using the FITC-Annexin V apoptosis detection kit-I (556547, BD Biosciences, San Jose, CA), followed by FACS analysis with annexin V and propidium iodide (PI).

Mouse cardiac fibroblasts were isolated from the cardiomyocyte-fibroblast mixture by centrifugation at a low speed of 20 g for 3 min. Cells on the top layer were collected and centrifuged at 300 g for 5 min. All fibroblasts derived from each mouse were collected on a 6-cm dish and cultured in high glucose DMEM containing 10% FBS and 1% penicillin-streptomycin. Cells from 2∼5 passages were used. Cardiac fibroblasts at about 70% confluence on a 6-well plate were starved for 24 h and then stimulated with or without different concentrations of ILC2 lysate (equivalent to 0, 10^3^, 10^4^, 2 × 10^4^ ILC2/ml) followed by treatment with or without TGF-β (transforming growth factor-β, 10 ng/mL, 14-8342-80, eBioscience) for 30 min. Cells were harvested for immunoblot analysis.

### 2.10 Mouse lymphocytes and apoptosis detection

Lymphocytes were isolated from mouse spleen by density gradient centrifugation with the LSM™ lymphocyte separation medium (0850494X, MP Biomedicals) according to the manufacturer’s instructions. Lymphocytes were stimulated using plate-bound rat anti-mouse CD3 monoclonal antibodies (5 µg/mL, 100223, BioLegend) and hamster anti-mouse CD28 monoclonal antibodies (3 µg/mL, 102121, BioLegend), and cultured in complete RPMI 1640 medium supplemented with 10% FBS and 1% penicillin-streptomycin at 37°C with 5% CO_2_. The lymphocytes were treated with different concentrations of ILC2 lysate (equivalent to 0, 2 × 10^3^, 2 × 10^4^ ILC2/mL), and with or without 100 µM H_2_O_2_ for 4 h before harvest. Cell apoptosis was detected using the FITC-Annexin V apoptosis detection kit-I (556547, BD Biosciences), followed by FACS analysis.

### 2.11 Immunoblot analysis

Bcl2, total Smas2/3, p-Smad-2/3, and cleaved caspase-3 expression in mouse cardiomyocytes or mouse cardiac fibroblasts were measured by immunoblot analysis. Proteins extracted from cardiomyocytes and fibroblasts were separated on 12% or 10% SDS-PAGE and then transferred to a polyvinylidene fluoride membrane. After blocking with 5% BSA for 2 h, the membrane was incubated with rabbit mAb Bcl-2 (1:1000, 3498S), rabbit anti-mouse total Smad-2 (1:1000, 5339S), rabbit anti-mouse p-Smad-2 (1:1000, 3108S), rabbit anti-mouse total Smad-3 (1:1000, 9523S, (all from Cell Signaling Technology, Inc. Danvers, MA), rabbit anti-mouse p-Smad-3 (1:1000, ab52903, Abcam, Cambridge, MA), rabbit anti-cleaved caspase-3 (1:1000, 9661S), and rabbit anti-mouse GAPDH (1:1000, 2118S, Cell Signaling Technology) at 4°C overnight followed by incubation with a HRP-conjugated secondary antibody (1:3000, G21234, Thermo Fisher Scientific) for 2 h at room temperature. The resulting signals were detected using the Amersham ECL Prime Western Blotting Detection Reagent (RPN2236, Fisher Scientific). GAPDH was used to ensure equal protein loading.

### 2.12 TUNEL, H&E, Sirius red, and immunofluorescent staining

The heart tissue was cut into 5-µm thickness sections and stored at −80°C for future use, including terminal deoxynucleotidyl transferase dUTP nick-end labelling (TUNEL), H&E, Sirius red, and immunofluorescent staining. The cell death in cultured cells was measured by TUNEL staining according to the manufacturer’s protocols (11684795910, Sigma-Aldrich). H&E staining was performed to measure the infarct size according to manufacturer’s instruction (HT110116, Sigma-Aldrich). Sirius red staining (365548-25G, Sigma-Aldrich) was used to detect and quantify red and orange collagen-I and green collagen-III on frozen heart sections according to our earlier studies.^25^ Immunofluorescent staining or co-staining was used to test donor ILC2 in recipient mouse heart, cardiomyocyte apoptosis, cardiac fibroblast apoptosis, IL33, IL4, IL5, Th1 cells, and Th2 cells.

Mouse heart frozen sections were fixed with cold acetone for 5 min followed with blocking nonspecific binding of secondary antibody by incubation with PBS supplemented with 5% BSA for 1 h at room temperature. The heart section was then stained with CD45.1 antibody (1:200, 1795-02, Southern Biotech, Birmingham, AL) for overnight, followed by anti-FITC-biotin antibody (1:400, B0287, Sigma-Aldrich) incubation for 1 h and Alexa Fluor™ 488 conjugated streptavidin (1:500, S11223, Fisher Scientific) for 30 min. To detect donor ILC2 expression of ILC2 markers, we stained heart sections with CD45.1 antibody (1:200, 1795-02, Southern Biotech) together with ST2 polyclonal antibody (1:200, PA5-20077, Invitrogen, Carlsbad, CA) or ICOS polyclonal antibody (1:100,313502, BioLegend) for overnight, followed by1 h incubation with the anti-FITC-biotin antibody (1:400, B0287, Sigma-Aldrich). Then relevant secondary antibody was used for another hour.

To detect myocardial cardiomyocyte apoptosis, cardiac fibroblast apoptosis, IL33, IL4, IL5, Th1 cells, and Th2 cells, the heart sections were stained with cleaved caspase-3 (1:200, 9661S, Cell Signaling Technology, Inc) and cardiac troponin T monoclonal antibody (1:500, MA5-12960, Thermo Fisher Scientific) or CD90 (Thy-1) monoclonal antibody (1:500, 14-0901-85, Thermo Fisher Scientific), IL33 polyclonal antibody (1:100, PA5-20398, Thermo Fisher Scientific), IL4 monoclonal antibody (1:100, ab11524, Abcam), IL5 polyclonal antibody (1:100, PA5-96761, Thermo Fisher Scientific), CD4 polyclonal antibody (1:100, PAB18320, Abnova, Walnut, CA) and IFN-γ monoclonal antibody (1:100, ab11524, Abcam), or IL4 monoclonal antibody (1:100, 505802, BioLegend), followed by 1 h incubation with relevant secondary antibody, including goat anti-rabbit, Alexa Fluor® 488, (1:500, A11008), goat anti-rat, Alexa Fluor® 488, (1:500, A11006), goat anti-mouse, Alexa Fluor® 555, (1:500, A21422), goat anti-rabbit, Alexa Fluor® 555, (1:500, A21428), and goat anti-rat, Alexa Fluor® 555, (1:500, A21434). Total nuclei were stained with DAPI. The sections were mounted and captured by a confocal microscope.

### 2.13 Statistical analysis

All mouse data were expressed as mean ± SEM and obtained and analyzed blindly. We used non-parametric Mann–Whitney *U* test followed by Bonferroni correction to compare two-group data that did not pass the normality test. Non-parametric Kruskal–Wallis test (one-way ANOVA on ranks) was used for all cell culture data analysis that contained multiple group comparisons and that did not pass the normality test. SPSS16 version was used for analysis and *P* < 0.05 was considered significant.

## 3. Results

### 3.1 ILC2 accumulate in mouse heart post-MI

**Figure 1:**
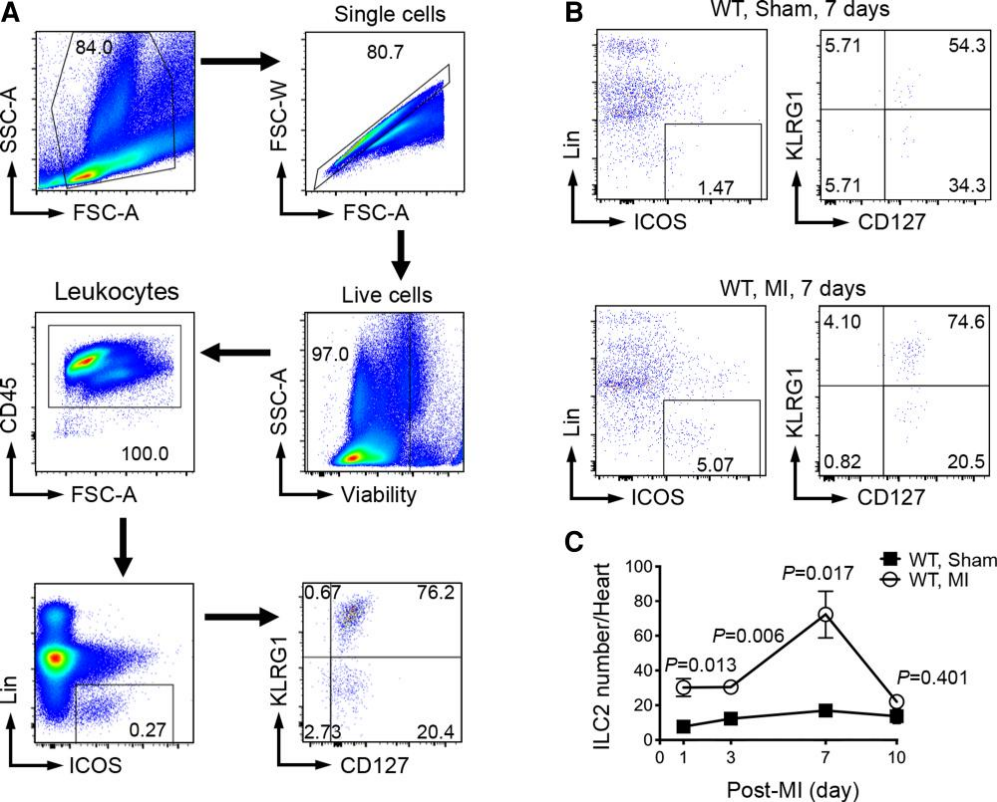
ILC2 accumulation in heart post-MI. (*A*) Establishment of FACS gating strategy to analyze mouse splenic ILC2 from WT mice at 3 days after IL33 treatment. (*B*) Representative FACS images of heart ILC2 analysis from WT sham and MI at 7 days post-MI. (*C*) FACS analysis of heart CD45^+^Lin^−^ICOS^+^CD127^+^KLRG1^+^ ILC2 counts in WT sham and WT MI mice at different days after surgery as indicated. Data are mean ± SEM and *n* = 3∼5 per group, non-parametric Mann–Whitney *U* test followed by Bonferroni correction.

To test the involvement of ILC2 in MI injury, we examined whether ILC2 accumulate in heart post-MI. ILC2 are defined as CD45^+^Lin^−^ICOS^+^CD127^+^KLRG1^+^ lymphocytes.^26^ The low quantity of these innate immune cells and their definition in expressing multiple cellular markers make it impossible and inaccurate to detect tissue ILC2 by immunofluorescent staining, although some studies tried to locate these cells in mouse atherosclerotic lesions.^17,27,28^ FACS analysis is commonly used. Due to the low quantity of ILC2, we used IL33 to induce ILC2 expansion^29,30^ to establish a valid FACS gating strategy to detect and quantify heart ILC2. WT mice (C57BL/6 background) were given IL33 (500 ng/day) for 3 days to enrich ILC2 *in vivo*. We then isolated splenocytes from IL33-treated mice and performed FACS to establish the CD45^+^Lin^−^ICOS^+^CD127^+^KLRG1^+^ ILC2 gating strategy (*Figure 1A*). To monitor ILC2 accumulation in post-MI heart, we produced LAD coronary artery permanent ligation-induced MI or sham operation in WT mice. At 1, 3, 7, and 10 days post-surgery, we collected heart and prepared single cells. At 1, 3, and 7 days post-surgery, we detected significantly more CD45^+^Lin^−^ICOS^+^CD127^+^KLRG1^+^ ILC2 in MI hearts than those in sham mouse hearts. Heart ILC2 peaked at 7 days post-MI but declined to the levels of those in sham mice at 10 days post-MI (*Figure 1B* and *C*). Increased ILC2 accumulation in post-MI hearts suggests their participation in MI.

### 3.2 Genetic deficiency of ILC2 aggravates post-MI cardiac dysfunction

**Figure 2:**
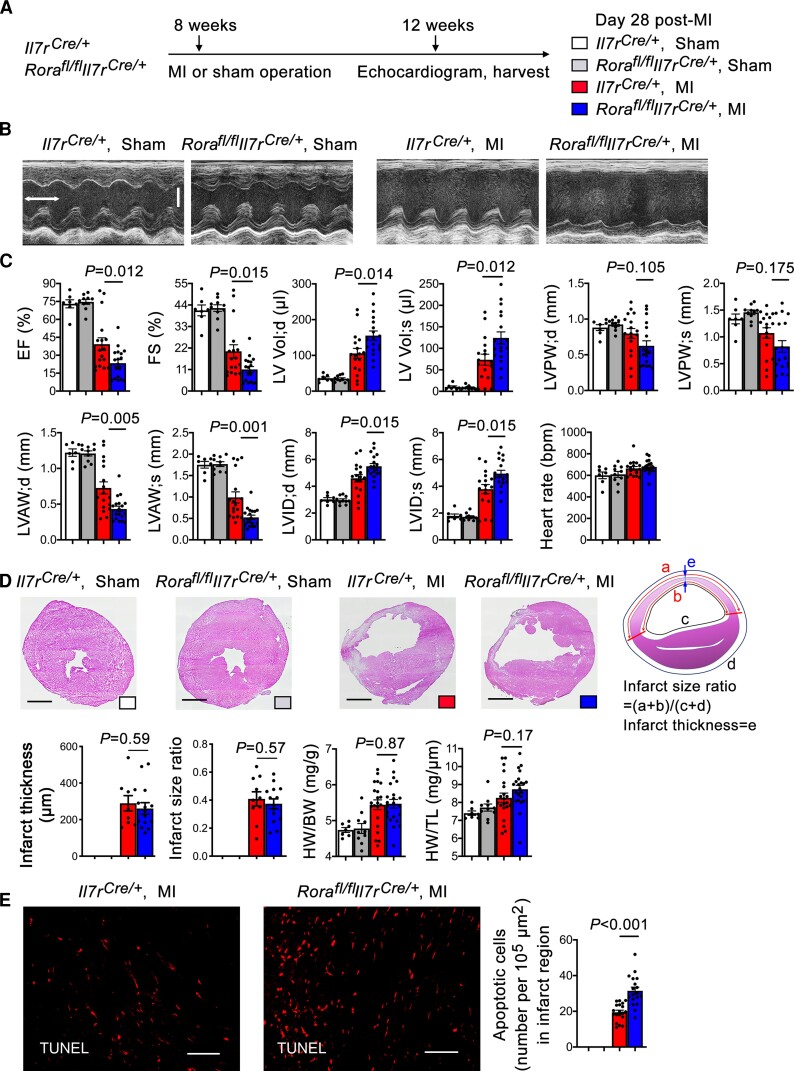
Deficiency of ILC2 aggravates cardiac dysfunction at 28 days post-MI. (*A*) Mouse surgery strategy. (*B*) Representative LV M-mode echocardiogram images of *Il7r^Cre/+^* and *Rora^fl/fl^Il7r^Cre/+^* mice at post-MI or sham as indicated (time stamp: 100 ms, scale: 1.7 mm). (*C*) Cardiac functions and heart rates in different groups of mice as indicated. (*D*) Infarct thickness and infarct size ratio and representative images (scale: 1.50 mm), calculation methods, BW/HW, and BW/TL. (*E*) TUNEL staining was used to detect apoptotic cells in hearts from different groups of mice (representative images on left, scale: 100 µm). Data are mean ± SEM. *n* = 7∼10 mice per sham group, *n* = 15∼20 mice per MI group, one-way ANOVA test followed by a post hoc Tukey’s test (*C* and *D*), or non-parametric Mann–Whitney *U* test followed by Bonferroni correction (*E*).

To examine a direct role of ILC2 in post-MI cardiac dysfunction, we produced MI or sham operation as outlined in *Figure 2A* in both ILC2-deficient *Rora^fl/fl^Il7r^Cre/+^* mice and *Il7r^Cre/+^* control mice that have been reported previously in mouse allergy models.^16^ We performed echocardiogram before harvest to assess the cardiac function and measured the heart weight (HW), body weight (BW), tibia length (TL), and infarct size at 28 days post-MI. The ratio of area at risk to LV area did not differ between *Rora^fl/fl^Il7r^Cre/+^* and *Il7r^Cre/+^* control mice post-MI, indicating that the ligation was performed reproducibly at the same level of the LAD coronary artery. At 28 days post-MI, the *Il7r^Cre/+^* mice with MI showed remarkably reduced cardiac function, as demonstrated by reduced EF% and FS%, increased end-diastole and end-systole LV volumes (LV Vol;d, and LV Vol;s), reduced end-diastole and end-systole LV anterior wall thickness (LVAW;d and LVAW;s), reduced end-diastole and end-systole LV posterior wall thickness (LVPW;d and LVPW;s), and increased LV end-diastole and end-systole internal diameter (LVID;d and LVID;s) compared with those of the sham groups. ILC2-deficient *Rora^fl/fl^Il7r^Cre/+^* mice, however, showed significantly further reduced EF% and FS%, increased LV Vol;d, and LV Vol;s, reduced LVAW;d and LVAW;s, and increased LCID;d and LVID;s (*Figure 2B* and *C*), indicating a beneficial role for ILC2 in post-MI heart. However, LVPW;d and LVPW;s, heart rate, infarct thickness and infarct size ratio measured by H&E staining, HW/BW and HW/TL ratios did not differ significantly between the *Rora^fl/fl^Il7r^Cre/+^* and *Il7r^Cre/+^* mice (*Figure 2C* and *D*). Sirius red staining was used to detect heart collagen-I (red and yellow colors) and collagen-III (green color) deposition.^31^ We also did not detect significant differences of collagen-I and collagen-III in the infarct, media, and remote regions between the groups (Supplementary material online, *Figure S1A*). These observations may explain the insignificant differences of HW/BW and HW/TL ratios between these mice (*Figure 2D*). While IL33 triggers ILC2 expansion,^29,30^ global deficiency of ILC2 may affect cardiac IL33 expression. Use of immunofluorescent staining did not show any difference in IL33 expression in the infarct, media, and remote regions between *Rora^fl/fl^Il7r^Cre/+^* and *Il7r^Cre/+^* mice (Supplementary material online, *Figure S1B*). TUNEL staining was used to detect cardiac cell death post-MI. Use of this method detected negligible cardiac cell death in sham-operated *Rora^fl/fl^Il7r^Cre/+^* and *Il7r^Cre/+^* mice, but the number of TUNEL-positive apoptotic cells was increased in post-MI hearts from *Il7r^Cre/+^* mice and increased further in post-MI hearts from *Rora^fl/fl^Il7r^Cre/+^* mice (*Figure 2E*).

Similar results were obtained when *Rora^fl/fl^Il7r^Cre/+^* and *Il7r^Cre/+^* mice were characterized at 1 day or 7 days post-MI. At 1 day post-MI, *Rora^fl/fl^Il7r^Cre/+^* mice also showed lower EF and FS and higher LV Vol; d and LV Vol; s than the *Il7r^Cre/+^* mice, although systolic and diastolic LVPW, LVAW, and LVID, heart rate, HW/BW, and HW/TL did not differ between the groups (Supplementary material online, *Figure S2A–D*). Yet, at this time point, we detected significantly thinner infarct and larger infarct size in *Rora^fl/fl^Il7r^Cre/+^* mice than in *Il7r^Cre/+^* mice (Supplementary material online, *Figure S2D*). Again, use of Sirius red staining did not detect any differences in infarct, media, and remote region collagen-I and collagen-III levels between the groups (Supplementary material online, *Figure S2E* and *F*). At 7 days post-MI, many more cardiac dysfunction variables became worse in *Rora^fl/fl^Il7r^Cre/+^* mice than in *Il7r^Cre/+^* mice, including lower EF and FS, higher LV Vol;d and LV Vol;s, lower LVPW;s, LVAW;d, and LVAW;s, and higher LVID;d and LVID;s. Different from those at 1 day or 28 days post-MI, *Rora^fl/fl^Il7r^Cre/+^* mice showed greatly thinner infarct thickness, larger infarct size, and higher HW/BW and BW/TL (Supplementary material online, *Figure S3A–D*). Consistent with these results, *Rora^fl/fl^Il7r^Cre/+^* mice showed much more infarct region collagen-I contents than the *Il7r^Cre/+^* mice, although collagen-I in media and remote region or collagen-III contents did not differ between the groups (Supplementary material online, *Figure S3E and F*). These observations suggest that the cardiac reparative role of ILC2 may vary at different time points post-MI.

### 3.3 ILC2 deficiency enhances the pro-inflammatory states in mouse heart post-MI

**Figure 3:**
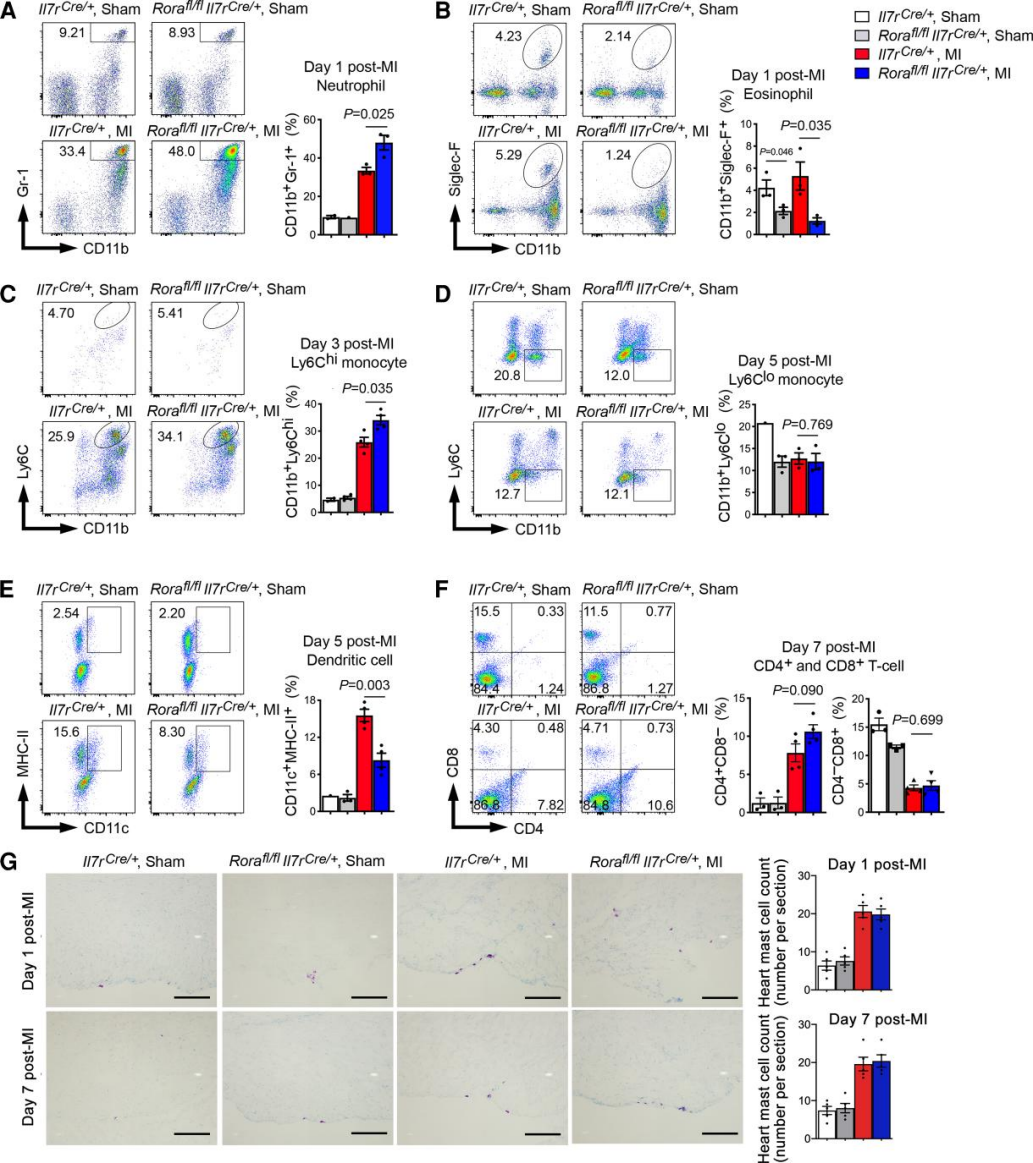
ILC2 deficiency facilitates acute accumulation of heart immune cells post-MI. (*A–F*) FACS analysis of heart CD45^+^CD11b^+^Gr-1^+^ neutrophils (1 day post-MI) (*A*), CD45^+^CD11b^+^Siglec-F^+^ EOS (1 day post-MI) (*B*), CD45^+^CD11b^+^Ly6C^hi^ monocytes (3 days post-MI) (*C*) CD45^+^CD11b^+^ Ly6C^lo^ monocytes (5 days post-MI) (*D*), CD45^+^MHC-II^+^CD11c^+^ dendritic cells (5 days post-MI) (*E*), and CD45^+^CD4^+^CD8^−^ and CD45^+^CD4^−^CD8^+^ T cells (7 days post-MI) (*F*). (*G*) Toluidine blue staining of cardiac mast cells. Representative images are shown to the left. FACS data were presented as percentage of total heart live CD45^+^ immune cells. Data are mean ± SEM. *n* = 4–6 mice per group, one-way ANOVA test followed by a post hoc Tukey’s test.

Inflammatory cell accumulation to the heart is a hallmark of inflammatory injury after MI.^32^ Exacerbated cardiac dysfunction in *Rora^fl/fl^Il7r^Cre/+^* mice relative to *Il7r^Cre/+^* mice post-MI suggests an adverse effect of ILC2 deficiency in post-MI heart inflammation. To examine this hypothesis, we performed FACS analysis of different types of inflammatory cells in the infarcted heart at a time course from 1, 3, 5, 7 days post-MI. At 1 day post-MI, ILC2 deficiency increased heart CD45^+^CD11b^+^Gr-1^+^ neutrophils but reduced heart CD45^+^CD11b^+^Siglec-F^+^ EOS (*Figure 3A* and *B*). At 3 days post-MI, ILC2 deficiency increased heart CD45^+^CD11b^+^Ly6C^hi^ monocyte content (*Figure 3C*). At 5 days post-MI, ILC2 deficiency did not affect heart CD45^+^CD11b^+^Ly6C^lo^ monocyte content, but significantly reduced heart CD45^+^CD11c^+^MHC-II^+^ DCs (*Figure 3D* and *E*). At 7 days post-MI, *Rora^fl/fl^Il7r^Cre/+^* mouse heart showed elevated CD4^+^ T cells, but not CD8^+^ T cells and such elevation did not reach statistical significance (*Figure 3F*). Of note, we detected significantly fewer EOS and Ly6C^lo^ monocytes in sham hearts from *Rora^fl/fl^Il7r^Cre/+^* mice than those from *Il7r^Cre/+^* mice (*Figure 3B* and *D*). Cardiac tissue toluidine blue staining showed that, at either 1 day or 7 days post-MI, myocardium mast cell counts did not differ between the groups (*Figure 3G*). As we will discuss later, deficiency of EOS and DC in *Rora^fl/fl^Il7r^Cre/+^* mouse heart may provide a mechanistic explanation of the ILC2 cardioprotective function in post-MI heart. At 28 days post-MI, however, heart inflammation becomes less important.^33^ Therefore, we assessed the inflammatory cell changes in the spleen instead. At this time point, we detected significantly reduced splenic Ly6C^lo^ monocytes and DC in *Rora^fl/fl^Il7r^Cre/+^* mice than in *Il7r^Cre/+^* mice. All other tested cells, including neutrophils, Ly6c^hi^ monocytes, CD4^+^, and CD8^+^ T cells, did not differ between the groups (Supplementary material online, *Figure S4*).

### 3.4 Induced ILC2 depletion also exacerbates post-MI cardiac dysfunction

**Figure 4:**
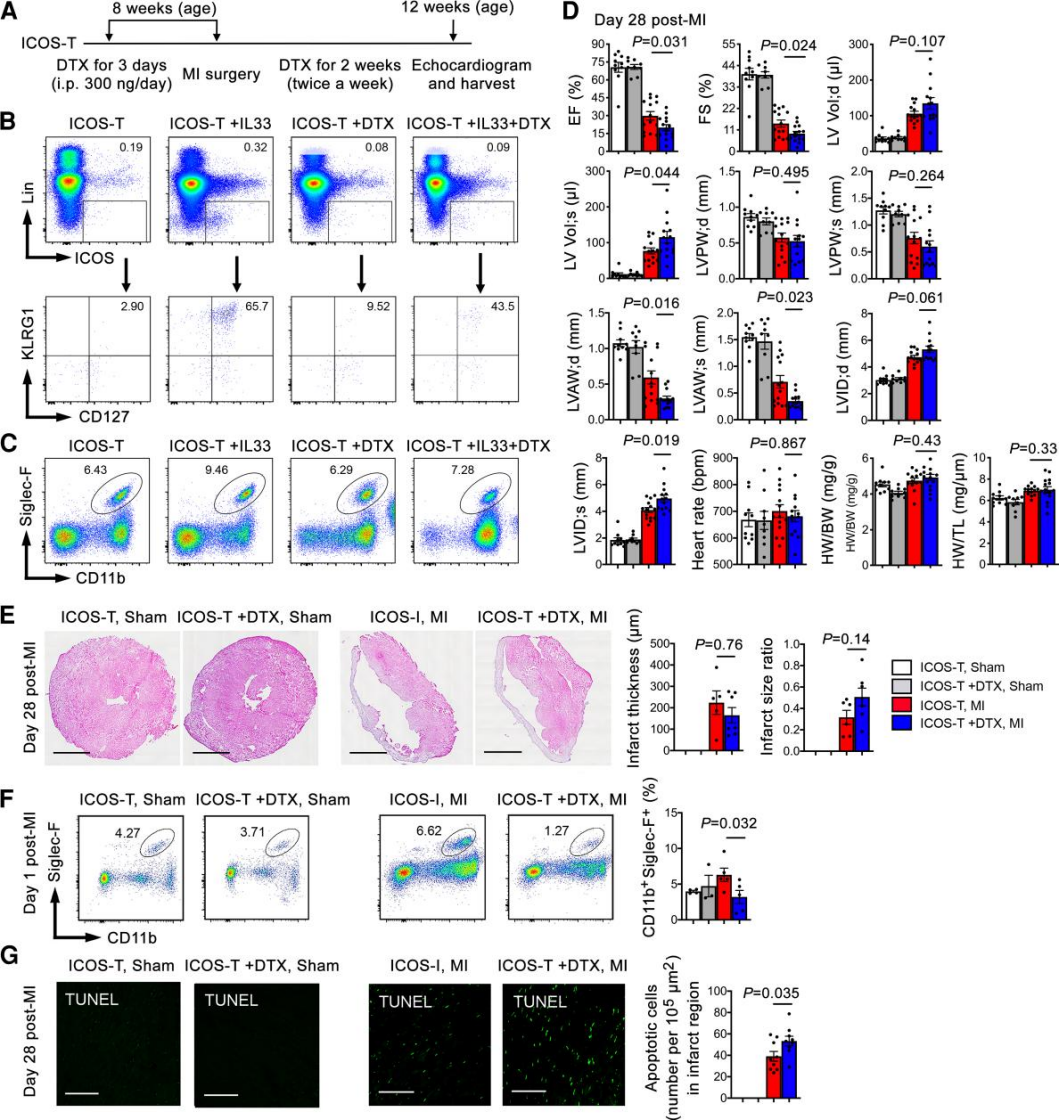
Induced ILC2 depletion exacerbates post-MI cardiac dysfunction. (*A*) Mouse surgery and treatment strategy. (*B/C*) Representative FACS analysis of splenic ILC2 and EOS in *ICOS-T* mice treated with or without IL-33 for 3 days to increase ILC2 production, and then with or without DTX injection for 3 days as indicated. (*D*) Cardiac functions, HW/BW, and HW/TL in *ICOS-T* mice with different treatments (without IL33 treatment) at 28 days post-MI. (*E*) Infarct thickness and infarct size ratio. (*F*) FACS analysis of heart CD45^+^CD11b^+^Siglec-F^+^ EOS at 1 day post-MI (without IL33 treatment). (*G*) TUNEL detected apoptotic cells in hearts from different groups of mice at 28 days post-MI or sham surgery. Representative images are shown to the left. Scales: 1.5 mm (*E*) and 100 µm (*G*). Data are mean ± SEM. *n* = 8∼10 mice per sham group, *n* = 13∼15 mice per MI group, one-way ANOVA test followed by a post hoc Tukey’s test (*D* and *F*) or non-parametric Mann–Whitney *U* test followed by Bonferroni correction (*E* and *G*).

To test further a cardioprotective role of ILC2 post-MI and to avoid possible confounding effects from ILC2 genetic deficiency, we produced MI and sham operation in ICOS-T mice that allow selective depletion of ILC2 by giving mice DTX as reported previously.^16^ We first treated ICOS-T mice with DTX (300 ng/mouse/day, i.p.) for 3 days to deplete ILC2. These mice were then subjected to MI surgery or sham operation and receive DTX twice a week (*Figure 4A*). ICOS-T mouse cardiac functions after MI or sham operation were assessed in 28 days. To ensure successful depletion of ILC2, we gave ICOS-T mice with or without IL33 for 3 days to boost ILC2 production and then treated these mice with and without DTX for 3 days. FACS analysis of splenocyte preparation demonstrated successful depletion of splenic CD45^+^Lin^−^ICOS^+^CD127^+^KLRG1^+^ ILC2 in ICOS-T mice treated with IL33 and DTX (*Figure 4B*). Under the same condition, IL33 or DTX did not affect splenic EOS contents (*Figure 4C*). As the *Rora^fl/fl^Il7r^Cre/+^* mice, DTX-induced ILC2 depletion in ICOS-T mice further impaired cardiac function, such as reduced EF and FS, and increased LV Vol;s, reduced LVAW;d and LVAW;s, and increased LVID;s, compared with those without DTX treatment (*Figure 4D*), although heart rate, HW/BW, HW/TL, infarct thickness, and infarct size ratio did not differ between the groups (*Figure 4D* and *E*). Like in the *Rora^fl/fl^Il7r^Cre/+^* mice, we also did not detect significant differences in infarct, media, and remote collagen-I and collagen-III contents (Supplementary material online, *Figure S5A*) or cardiac IL33 levels (Supplementary material online, *Figure S5B*) at this time point. Due to the small numbers of heart ILC2 without IL33 treatment, we were unable to accurately quantify heart ILC2 by FACS in ICOS-T mice with or without DTX treatment or MI production. Splenic immune cell FACS analysis showed that DTX treatment did not affect neutrophil, Ly6C^hi^ and Ly6C^lo^ monocyte, DC, EOS, and CD4^+^ and CD8^+^ T cell contents between the sham and MI ICOS-T mice, whether these mice were treated with or without DTX for 3 days before MI or sham surgery (Supplementary material online, *Figure S6*). However, like in the *Rora^fl/fl^Il7r^Cre/+^* mice, DTX-induced ILC2 depletion reduced heart EOS accumulation at 1 day post-MI in ICOS-T mice treated with DTX, as determined by FACS analysis of heart total single cell preparation (*Figure 4F*). ILC2 depletion also increased cardiac cell apoptosis at 28 days post-MI as determined by heart section TUNEL staining (*Figure 4G*). These observations further support a cardioprotective role of ILC2 in post-MI heart. ILC2 may protect heart from MI-induced cardiac cell apoptosis.

### 3.5 ILC2 protect heart from post-MI injury by the IL5, EOS, and DC mechanism

**Figure 5:**
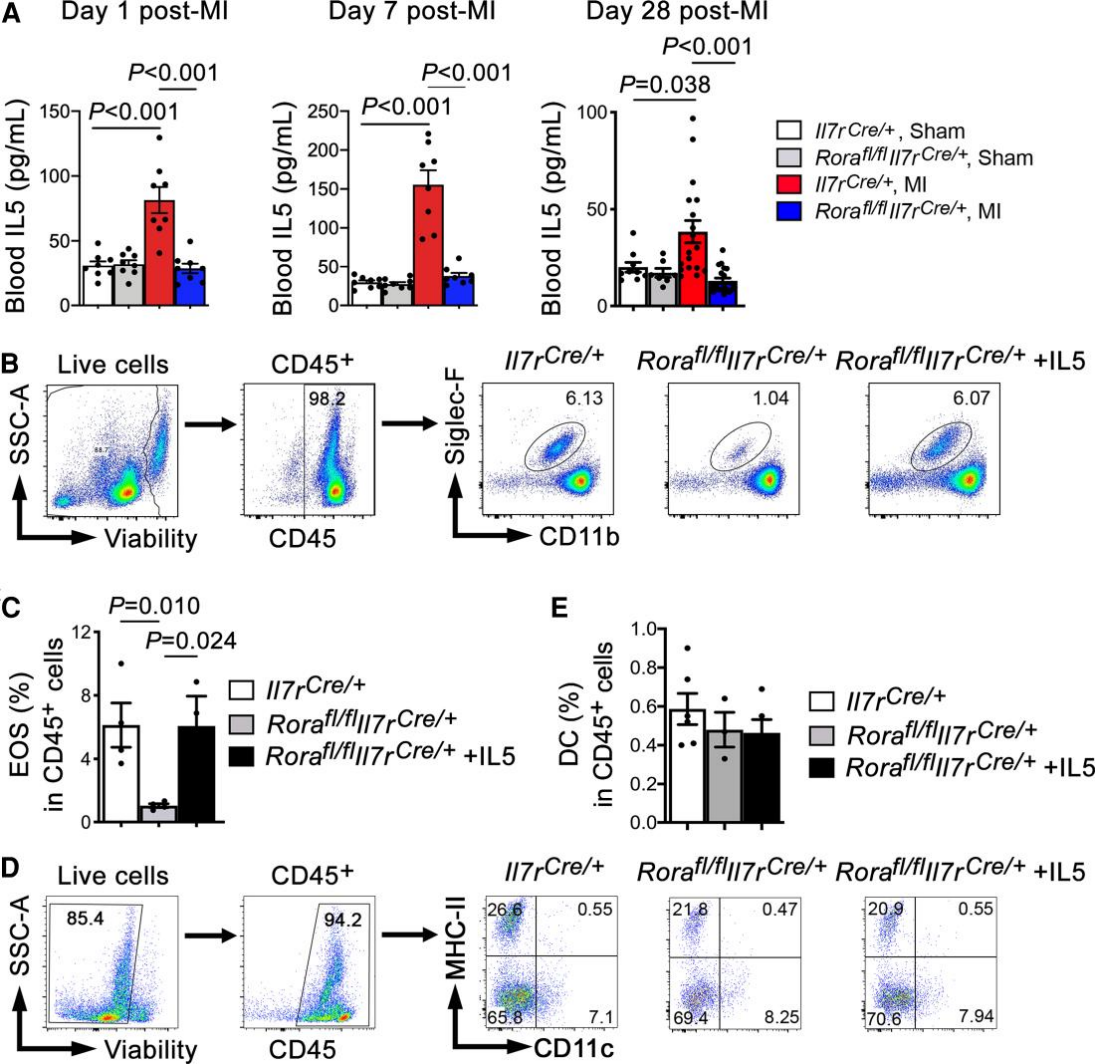
IL5 reverses splenic depletion of EOS but not DC in ILC2-deficient *Rora^fl/fl^Il7r^Cre/+^* mice. (*A*) ELISA detected mouse plasma IL5 levels from different groups of mice at 1, 7, and 28 days post-MI or sham surgery as indicated. *n* = 8∼18 mice per group. (*B–E*) Gating strategy and representative FACS images to detect mouse splenic EOS (*B/C*) and DC contents (*D/E*) in *Il7r^Cre/+^* and *Rora^fl/fl^Il7r^Cre/+^* mice and those received IL5 (40 ng/mouse/day) for 12 days. *n* = 4∼6 mice per group, one-way ANOVA test followed by a post hoc Tukey’s test.

ILC2 are the major source of IL5,^34^ an essential regulator of EOS development and maturation in the bone-marrow. We recently reported a cardioprotective role of EOS in post-MI hearts.^18^ Therefore, we hypothesized that ILC2 deficiency leads to systemic deficiency of IL5 and EOS. The cardiprotective role of ILC2 in post-MI hearts (*Figure 2A–E*) is likely mediated indirectly by IL5 and EOS. To test this hypothesis, we first measured plasma IL5 levels in *Rora^fl/fl^Il7r^Cre/+^* and *Il7r^Cre/+^* mice at 1, 7, and 28 days post-MI. Relative to the sham-operated *Il7r^Cre/+^* mice, MI production increased plasma IL5 levels in *Il7r^Cre/+^* mice. Such induction was blunted in *Rora^fl/fl^Il7r^Cre/+^* mice (*Figure 5A*). This may explain why *Rora^fl/fl^Il7r^Cre/+^* mice showed EOS deficiency regardless whether these mice underwent sham operation or MI injury (*Figure 3B*). In spleens, we also detected EOS deficiency in *Rora^fl/fl^Il7r^Cre/+^* mice. EOS levels were fully recovered after *Rora^fl/fl^Il7r^Cre/+^* mice received IL5 treatment for 12 days (*Figure 5B* and *C*). ILC2- or EOS-derived Th2 cytokines contribute to the development of DC.^7,22,23^ Lack of these cytokines may have caused CD11c^+^MHC-II^+^ DC deficiency in *Rora^fl/fl^Il7r^Cre/+^* mouse heart and spleens post-MI (*Figure 3E*; Supplementary material online, *Figure S4*). Yet, ILC2 deficiency or recombinant IL5 treatment for 12 days did not change splenic DC contents in *Rora^fl/fl^Il7r^Cre/+^* mice (*Figure 5D* and *5E*), suggesting an IL5-independent mechanism of DC deficiency in *Rora^fl/fl^Il7r^Cre/+^* mouse heart post-MI.

**Figure 6:**
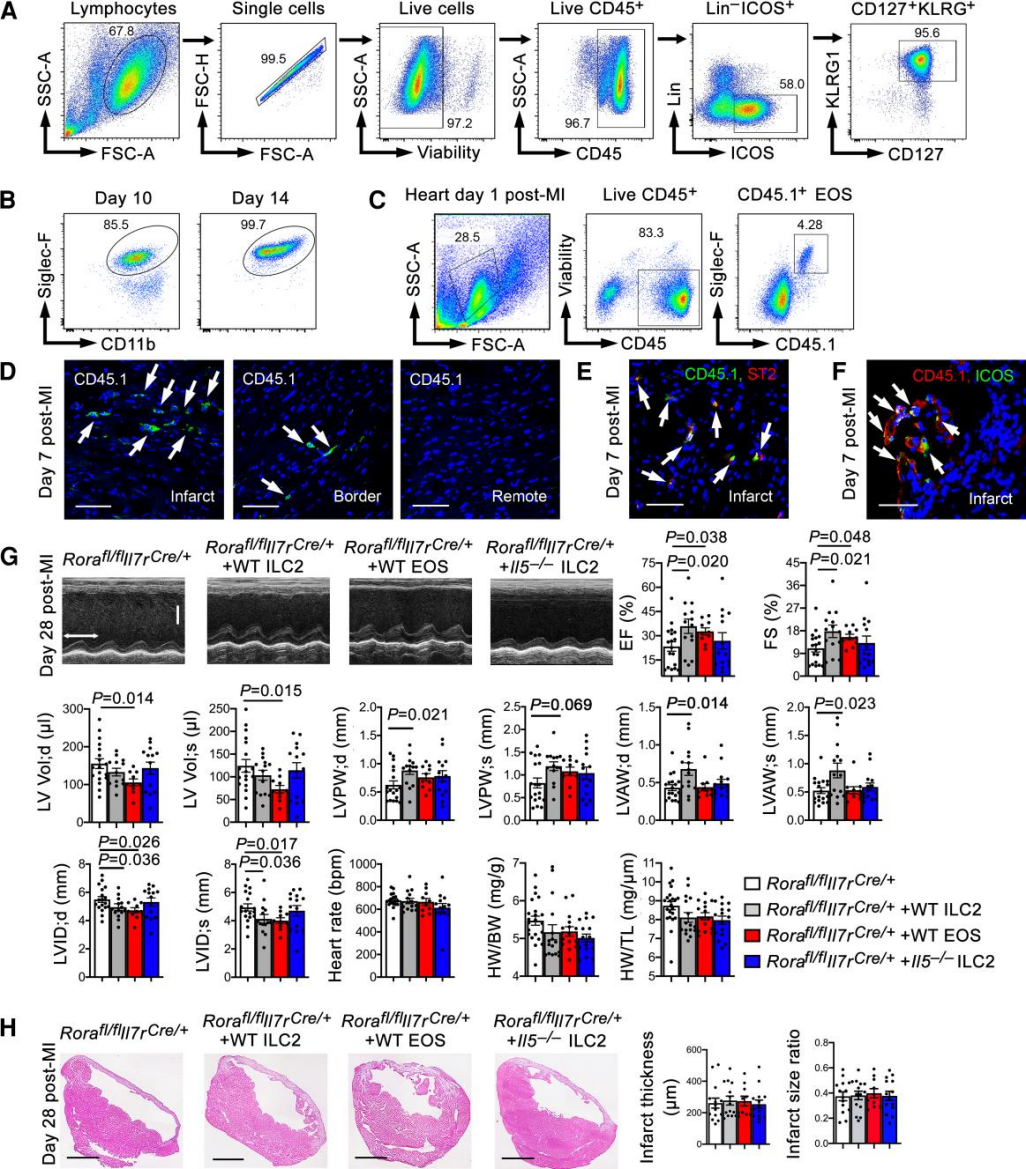
Reconstitution of ILC2 or EOS from WT mice protects mice from post-MI cardiac dysfunction. (*A*) Sorting strategy to purify mouse CD45^+^Lin^−^ICOS^+^CD127^+^KLRG1^+^ ILC2 from mouse splenocytes. (*B*) FACS detected the purity of *in vitro* prepared EOS at 10 and 14 days after bone-marrow cell differentiation. (*C*) FACS detected donor CD45.1^+^ EOS in recipient mouse heart at 1 day post-MI. (*D–F*) Immunofluorescent staining and confocal imaging to detect CD45.1^+^ donor ILC2 in infarct, border, and remote regions (*D*), CD45.1^+^ST2^+^ donor ILC2 in infarct region (*E*), and CD45.1^+^ICOS^+^ donor ILC2 in infarct region (*F*) in *Rora^fl/fl^Il7r^Cre/+^* recipient mouse heart at 7 days post-MI. Scale: 40 µm. (*G*) Representative LV M-mode echocardiogram images, cardiac functions, HW/BW, and HW/TL from *Rora^fl/fl^Il7r^Cre/+^* mice and those received adoptive transfer of WT ILC2, WT EOS, and *Il5*^−*/*−^ ILC2 at 28 days post-MI (time stamp: 100 ms, scale: 1.7 mm). (*H*) Infarct thickness and infarct size ratio at 28 days post-MI. Representative H&E staining images are shown to the left. Scale: 1.50 mm. Data are mean ± SEM. *n* = 10∼14 mice per group, one-way ANOVA test followed by a post hoc Tukey’s test.

To test the hypothesis that ILC2 play a cardioprotective role indirectly by producing IL5 and promoting EOS development, we performed adoptive transfer of ILC2 and EOS from WT mice, and ILC2 from IL5-deficient (*Il5*^−*/*−^) mice to *Rora^fl/fl^Il7r^Cre/+^* recipient mice, followed by producing MI in recipient mice. Donor CD45^+^Lin^−^ICOS^+^CD127^+^KLRG1^+^ ILC2 from WT and *Il5*^−*/*−^ mice were isolated from mouse mesenteric lymph nodes and spleens by cell sorting after mice received IL33 for 3 days. IL33 was used only for enriching ILC2 in donor mice.^29,30^ The sorting strategy is outlined in *Figure 6A*. EOS were obtained from bone-marrow cell differentiation as reported.^18,21,24^ After 14 days, the purity of *in vitro* differentiated CD11b^+^Siglec-F^+^ EOS reached to above 99.5% as verified by FACS (*Figure 6B*). To monitor donor EOS and ILC2 targeting to the heart in recipient mice after adoptive transfer, we used donor EOS and ILC2 from CD45.1 transgenic mice. FACS revealed CD45.1^+^ donor EOS in recipient mouse heart at 1 day post-MI (*Figure 6C*). Although low numbers of ILC2 did not allow FACS detection, we performed immunofluorescent staining using anti-CD45.1 antibody to detect donor ILC2 from the CD45.1 transgenic mice and showed their localization mainly in the infarct region, few donor ILC2 in the border region, but negligibly in the remote region at 7 days post-MI (*Figure 6D*). Earlier studies used bone-marrow cell transfer, examined donor ILC2 in mouse heart at 1 month post-MI when myocardial inflammatory cell accumulation was minimal, and claimed that donor ILC2 did not target to the heart.^35^ Here we used freshly prepared mature ILC2 as donor cells. To confirm that these donor ILC2 from the CD45.1 transgenic mice remained their ILC2 phenotypes, we performed immunofluorescent double staining and confocal imaging and showed that those CD45.1^+^ donor cells in infarcted heart expressed ILC2 markers ST2 (*Figure 6E*) and ICOS (*Figure 6F*). To the *Rora^fl/fl^Il7r^Cre/+^* recipient mice, donor ILC2 and EOS from WT mice increased significantly the EF and FS and reduced LVID;d and LVID;s. Donor EOS also reduced significantly LV Vol;d and LV Vol;s and donor ILC2 increased end-diastole and end-systole LVPW and LVAW. In contrast, ILC2 from *Il5*^−*/*−^ mice failed to change any of these cardiac function variables (*Figure 6G*). As in the *Rora^fl/fl^Il7r^Cre/+^* and *Il7r^Cre/+^* mice, with or without adoptive transfer of ILC2 or EOS did not affect HW/BW and HW/TL ratio and infarct thickness or infarct size (*Figure 6G* and *H*) at this time point. Adoptive transfer of DC from WT mice also increased EF and FS, reduced LV Vol;d and LV Vol;s, and increased infarct thickness in *Rora^fl/fl^Il7r^Cre/+^* recipient mice at 7 and 28 days post-MI (Supplementary material online, *Figure S7A–D*). At 7 days post-MI, donor DC also increased LVPW;s, LVAW;d, LVAW;s, reduced LVID;d and LVID;s, reduced infarct size, and HW/BW and HW/TL ratios (Supplementary material online, *Figure S7A–D*). Consistent with these observations, donor DC reduced infarct region collagen-I but not collagen-III at 7 days post-MI. At 28 days post-MI, donor DC showed no effect on collagen deposition at infarct, media, or remote regions (Supplementary material online, *Figure S7E–H*). Together, these observations support the hypothesis that ILC2 play an indirect role in protecting heart from MI injury and improving post-MI cardiac function by producing IL5 and other molecules that control EOS and DC development or accumulation in infarcted heart.

### 3.6 ILC2 affect cardiac cell activity *via* an indirect mechanism

**Figure 7:**
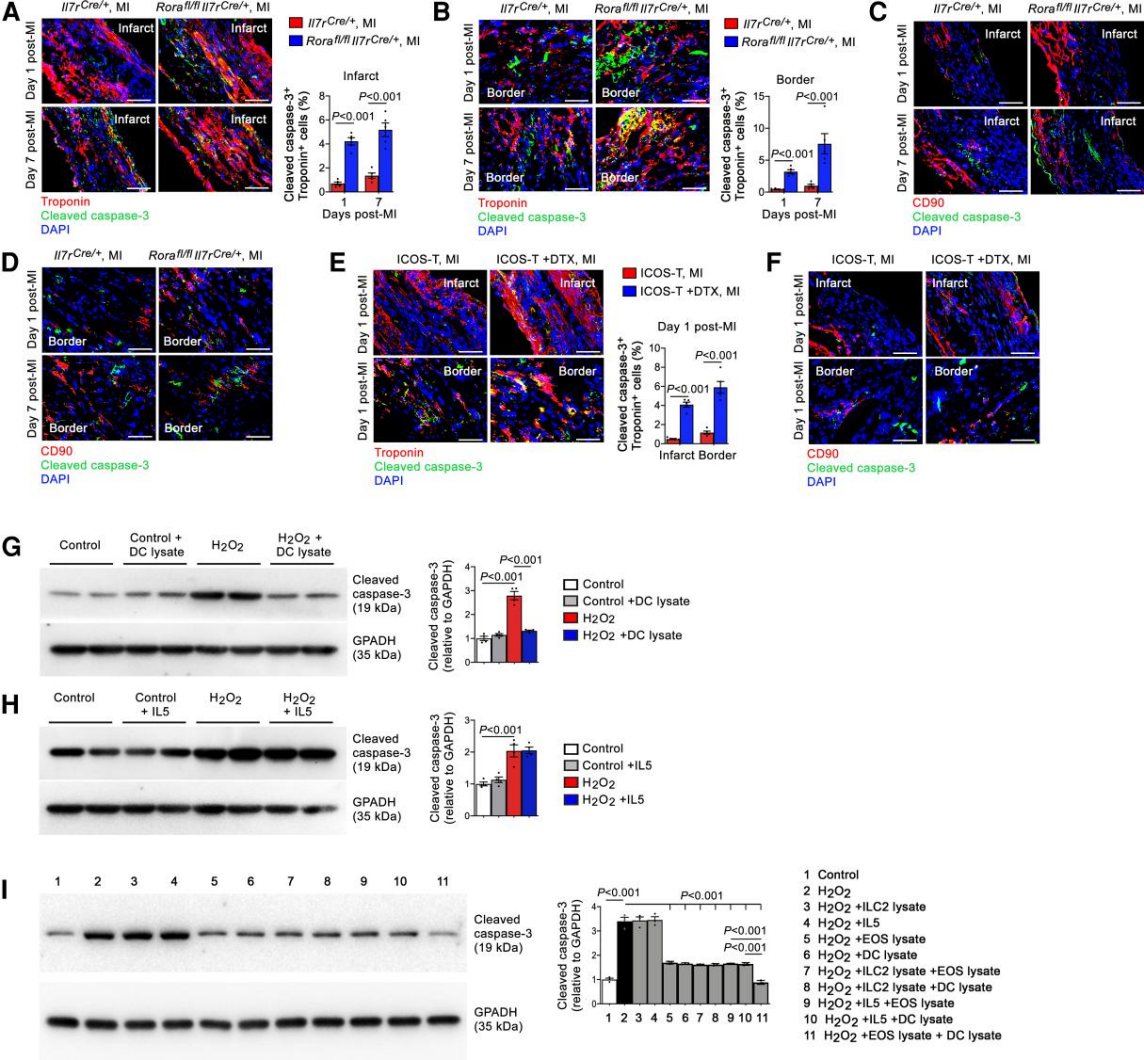
ILC2 and DC activity on cardiac cell apoptosis. (*A–D*) Immunofluorescent staining and confocal imaging of troponin and cleaved caspase-3 double positive apoptotic cardiomyocytes or CD90 and caspase-3 double positive apoptotic cardiac fibroblasts in the infarct (*A/C*) and border (*B/D*) regions from *Il7r^Cre/+^* and *Rora^fl/fl^Il7r^Cre/+^* mice at 1 or 7 days post-MI. (*E/F*) Apoptotic cardiomyocytes (*E*) and cardiac fibroblasts (*F*) in ICOS-T mice after 1 day post-MI. Scale: 50 µm. (*G–I*) Immunoblot analysis of cleaved caspase-3 in cardiomyocytes after H_2_O_2_-induced apoptosis with or without DC lysate (*G*), IL5 (*H*), or different combinations of ILC2 lysate, IL5, EOS lysate, and DC lysate as indicated (*I*). Data are mean ± SEM from five mice per group (*A–F*) or four independent experiments (*G–I*), non-parametric Mann–Whitney *U* test followed by Bonferroni correction (*A, B* and *E*), or one-way ANOVA test followed by a post hoc Tukey’s test (*G–I*).

Cardiac cell death and collagen synthesis contribute to post-MI cardiac dysfunction.^36,37^ We have previously shown that the apoptotic cells in the myocardium in post-MI mice are mostly cardiomyocytes.^18^ Immunofluorescent double staining and confocal imaging showed that, at both 1 and 7 days post-MI, troponin and cleaved caspase-3 double positive apoptotic cardiomyocyte contents increased in *Rora^fl/fl^Il7r^Cre/+^* mice in both the infarct and border regions (*Figure 7A* and *B*). In contrast, we saw negligible CD90 and cleaved caspase-3 double positive apoptotic cardiac fibroblasts in *Il7r^Cre/+^* or *Rora^fl/fl^Il7r^Cre/+^* mice at both time points (*Figure 7C* and *D*). DTX-induced ILC2-depleted ICOS-T mice yielded the same results. ILC2 depletion increased infarct and border region troponin and cleaved caspase-3 double positive apoptotic cardiomyocytes (*Figure 7E*), but negligible CD90 and cleaved caspase-3 double positive apoptotic cardiac fibroblasts were detected (*Figure 7F*). To test whether ILC2 contributed directly or indirectly to cardiomyocyte apoptosis, we induced mouse cardiomyocyte apoptosis with H_2_O_2_. Both Bcl-2 immunoblot and annexin V-PI (propidium iodide) FACS analyses showed that addition of different doses of ILC2 lysates did not affect H_2_O_2_-induced cardiomyocyte apoptosis (Supplementary material online, *Figure S8A* and *B*). When splenic lymphocytes were used, H_2_O_2_ also induced lymphocyte apoptosis. Different doses of ILC2 lysates again did not affect lymphocyte apoptosis (Supplementary material online, *Figure S8C*). These observations from cultured cardiomyocytes and lymphocytes suggest that the elevated cardiac cell death in post-MI heart from *Rora^fl/fl^Il7r^Cre/+^* mice (*Figures 2E*, *7A*, and *7B*) and DTX-treated ICOS-T mice (*Figures 4G* and *7E*) was not a direct role of ILC2.

ILC2 and EOS are important sources of Th2 cytokines^7^ that contribute to the development or differentiation of EOS and DC.^22,23,38^ Immunofluorescent staining showed that Th2 cytokines IL4 and IL5 accumulated mostly in the border region at 1, 7, and 28 days post-MI, but much less in the infarct region or negligible in the remote region (not shown). ILC2 deficiency in *Rora^fl/fl^Il7r^Cre/+^* mice blunted IL4 and IL5 productions (Supplementary material online, *Figure S9A* and *B*). When the myocardial sections were stained for CD4 together with Th1 cytokine IFN-γ or Th2 cytokine IL4, we found that ILC2 deficiency did not affect border region CD4^+^IFN-γ^+^ Th1 cell contents, but significantly reduced border CD4^+^IL4^+^ Th2 cells or increased the Th1/Th2 ratios (Supplementary material online, *Figure S10A* and *B*). We recently reported a role for EOS in blocking cardiomyocyte apoptosis.^18^ Here we showed that DC acted the same as EOS in inhibiting H_2_O_2_-induced cleaved caspase-3 production in mouse cardiomyocytes (*Figure 7G*). In contrast, recombinant IL5 did not exert this activity (*Figure 7H*). Therefore, only EOS and DC but not ILC2 or IL5 showed the activity to block cardiomyocyte apoptosis. Consistent with this conclusion, EOS and DC showed synergistic inhibitory activity against H_2_O_2_-induced cardiomyocyte apoptosis. ILC2 or IL5 did not affect the activity of EOS or DC on cardiomyocyte apoptosis (*Figure 7I*).

ILC2 deficiency (*Figures 2D*, *6G*, and *6H*) or DTX-induced ILC2 depletion (*Figure 4D* and *E*) did not affect HW/BW, HW/TL, infarct size, infarct thickness, or heart collagen deposition post-MI (Supplementary material online, *Figure S1A and S5A*) at 28 days post-MI. However, at 7 days post-MI, *Rora^fl/fl^Il7r^Cre/+^* mice showed thinner infarct thickness, larger infarct size, higher HW/BW and HW/TL ratios, and higher infarct collagen-I content than those of *Il7r^Cre/+^* control mice (Supplementary material online, *Figure S3C* and *F*). To test a role for ILC2 in cardiac fibroblast activation, we cultured mouse cardiac fibroblasts and treated cells with TGF-β with and without different doses of ILC2 lysates. Different doses of ILC2 lysate did not affect TGF-β-induced p-Smad2 and p-Smad3 signaling (Supplementary material online, *Figure S11*).

## 4. Discussion

Inflammatory cell accumulation is essential to post-MI cardiac remodeling and heart function.^39,40^ Here, we report an essential but indirect role of ILC2 in protecting mouse heart from MI injury by producing IL5 and possibly other untested molecules that promote the development and accumulation of EOS and DC in infarcted heart. This conclusion is supported by the finding that genetic deficiency or induced depletion of ILC2 exacerbated cardiac dysfunction post-MI. ILC2 deficiency reduced heart and splenic EOS and DC contents in *Rora^fl/fl^Il7r^Cre/+^* mice that underwent MI injury. Cardiac cell death was increased in *Rora^fl/fl^Il7r^Cre/+^* mice and in ICOS-T mice after DTX-induced ILC2 depletion post-MI. Only EOS and DC but not ILC2 or IL5 protected H_2_O_2_-induced cardiomyocyte apoptosis. Consistent with reduced heart EOS and DC counts, plasma IL5 levels and cardiac IL5 and IL4 were blunted in *Rora^fl/fl^Il7r^Cre/+^* mice post-MI. IL5 administration fully reversed splenic EOS counts in *Rora^fl/fl^Il7r^Cre/+^* mice, although IL5 did not directly affect splenic DC counts in *Rora^fl/fl^Il7r^Cre/+^* mice.

Although not all tested in this study, ILC2 may contribute to the post-MI cardiac reparative mechanism by affecting the pathobiology of not only EOS and DC but also many other immune cells, such as Ly6C^lo^ monocytes and B lymphocytes. *Il7r^Cre/+^* mice showed reduced heart Ly6C^lo^ monocytes post-MI. Different from the inflammatory Ly6C^hi^ monocytes, these reparative Ly6C^lo^ monocytes may preferentially differentiated into M2 macrophages that participate in post-MI heart late phase cardiac repair, although there is currently no clear distinction between the roles of Ly6C^hi^ and Ly6C^lo^ monocytes in post-MI cardiac function and Ly6C^lo^ monocytes have been considered from Ly6C^hi^ monocyte differentiation.^41,42^ ILC2 also produce IL5 to trigger B1a cell proliferation and natural IgM production.^13,14^ B1a cells secrete IgM natural antibody that reduces atherogenesis.^43^ Reduced plasma IL5 in *Rora^fl/fl^Il7r^Cre/+^* mice may lead to reduced B1a cells and plasma IgM, thereby promoting infarct expansion and LV dysfunction post-MI.^44^ Our earlier studies showed that inflammatory cells accumulate in mouse infarcted hearts in an orderly fashion. Neutrophils and Ly6C^hi^ monocytes peaked at 1 day post-MI, EOS peaked at 3 days post-MI, but Ly6C^lo^ monocytes and CD8^+^ T cells peaked at 7 days post-MI.^18^ Here we showed that ILC2 also peaked at 7 days post-MI. Although this study did not test the role of neutrophils or monocytes, neutrophils express chemokines CCL2 (CC chemokine ligand 2), CCL3, CXCL8 (C-X-C motif chemokine ligand 8) and monocytes express CXCL16 and CCL1.^45–47^ Many of these chemokines are known to mediate ILC2 migration.^48,49^ It is possible that these earlier arrivers in the infarcted heart may produce these chemokines to drive the accumulation of consequent ILC2, a hypothesis that merits further investigation.

We have recently reported that EOS protected MI-induced cardiac dysfunction by releasing IL4 and cationic protein mEar1 to protect cardiomyocytes from hypoxia or H_2_O_2_-induced apoptosis and block TGF-β-induced p-Smad2/3 signaling and collagen production.^18^ In MI patients, reduced myocardial DC are associated with impaired cardiac repair and development of MI rupture.^50^ In mice, administration of infarct tissue lysate-primed DC activates MI tissue reparative regulatory T cells and elicits MI reparative macrophage production, resulting improved post-MI cardiac function.^51^ This study proposed a hypothesis that ILC2 repair MI-induced heart injury indirectly via EOS and DC. The observation that impaired post-MI cardiac function in ILC2-deficient *Rora^fl/fl^Il7r^Cre/+^* mice was partially recovered by giving mice donor EOS or DC support this hypothesis. ILC2 deficiency or depletion led to EOS and DC deficiency in the heart with concurrent increase of cardiac cell death. Both EOS and DC, but not ILC2 or IL5 alone protected mouse cardiomyocytes from apoptosis. Therefore, EOS, DC, and possibly other untested cell types accounted for cardiomyocyte death protection. IL5 mediates EOS development in the bone-marrow.^34^ Reduced EOS in *Rora^fl/fl^Il7r^Cre/+^* mice were fully recovered after mice received recombinant IL5. Yet, IL5 did not affect splenic DC in *Rora^fl/fl^Il7r^Cre/+^* mice. Earlier studies showed that IL5 blocked GM-CSF-induced DC differentiation from bone-marrow culture. In contrast, IL4 reversed this inhibitory effect of IL5.^22,23^ EOS appear in infarcted heart at 1 day post-MI and peaked at 3 days post-MI,^18^ but DC appear in infarcted heart much later.^52^ Earlier arrivers EOS may produce IL4 and other untested molecules to support DC accumulation and *in situ* differentiation.^53,54^ a hypothesis that was not tested in this study.

Another interesting finding is that we did not detect significant differences in HW/BW, HW/TL, collagen deposition, infarct thickness, and infarct size ratios between *Rora^fl/fl^Il7r^Cre/+^* and *Il7r^Cre/+^* mice or between the ICOS-T mice that were treated with or without DTX when mice were analyzed at 28 days post-MI. These observations agree with the finding that ILC2 did not affect TGF-β-induced cardiac fibroblast activation. However, when *Rora^fl/fl^Il7r^Cre/+^* and *Il7r^Cre/+^* mice were analyzed at 7 days post-MI, *Rora^fl/fl^Il7r^Cre/+^* mice showed significantly higher HW/BW, HW/TL, infarct size ratios, and infarct region collagen-I deposition and thinner infarct thickness than those of *Il7r^Cre/+^* mice. We do not have direct evidence to suggest a role for ILC2 in collagen deposition. The results from this study showed that ILC2 peaked at 7 days post-MI and dropped to the baseline after 10 days post-MI. It is possible that ILC2 indirectly affect fibrotic protein expression and regulation at this peak time point, when the influence of ILC2 on cardiac fibrosis was maximized, a hypothesis requires further investigation.

Together, this study used the permanent ligation-induced MI model to establish a beneficial function of ILC2 in infarcted hearts partially by producing IL5 and possibly other untested Th2 cytokines that promote the development and differentiation of EOS and DC as an indirect mechanism to protect mice from post-MI cardiac dysfunction. This experimental model involves extensive cardiac inflammatory cell accumulation and cardiac remodeling. The results from this study shed new mechanistic light on the modulation of inflammation during the healing of myocardial ischemic injury. Stimulation of ILC2 growth or IL5 expression from these cells, such as IL2 and IL33 therapies that were reported recently,^55^ may benefit patients with MI injury.

## 5. Study limitations

As we discussed above, in addition to EOS and DC, this study did not test the role of other possible cell populations that might be affected by ILC2 and play a similar role to that of EOS and DC in infarcted heart, such as Th2 and B1a cells. Here we showed a similar role of EOS and DC in mitigating cardiac function post-MI, but this study did not explore how ILC2 affect DC differentiation. We only showed that ILC2-derived IL5 plays no role in DC development. Earlier studies demonstrated that ILC2 use IL13 to control DC differentiation.^56^ In turn, DC activate ILC2 and increase Th2 cytokine production.^57^ Reduced myocardial IL4 and IL5 levels and CD4^+^IL4^+^ Th2 cell contents in ILC2-deficient *Rora^fl/fl^Il7r^Cre/+^* mice post-MI support a hypothesis that reduced DC in these mice may contribute to these cardiac phenotypes in injured heart. Due to the small population of ILC2 in the heart and lack of sufficient source of this cell type or cell line from humans, this study did not test the role of ILC2 in human heart post-MI or the pathobiology of cultured human ILC2 on cardiac cells. As IL5, IL4, or IL13 are not specific to ILC2, there is no simple biomarker besides FACS characterization that can be used to monitor the changes of ILC2 in human heart or in the circulation following acute MI. Besides mouse studies and primarily culture mouse cells, we currently do not have any information from humans regarding the role of ILC2 in cardiac injury.

## Supplementary material

Supplementary material is available at *Cardiovascular Research* online.

## Authors’ contributions

T.L., J.L., J.L., Y.Z., Z.D., S.L., M.W., Q.H., P.F., J.D., Z.M., and S.Z. performed all in vitro and in vivo studies. A.N.J.M. provided the *Rora^fl/fl^Il7r^Cre/+^*, *Il7r^Cre/+^*, and *Icos^fl−DTR−fl/+^Cd4^Cre/+^* mice. G.-P.S. and J.G. designed the experiments. G-P.S. drafted the manuscript. G.-P.S., D.L., A.N.J.M., M.N., P.L., and J.G. helped critical reading and editing.

## Supplementary Material

cvac144_Supplementary_Data

## Data Availability

The data underlying this article will be shared on reasonable request to the corresponding author.

## Notes

Translational perspective ILC2 produces inflammatory molecules to control adaptive and innate immunities. This study demonstrates a cardiac reparative role for ILC2 in mouse post-MI heart by producing IL5 and possibly other untested molecules to affect eosinophil and dendritic cell development and accumulation in infarcted heart where these cells protect cardiomyocytes from apoptosis. ILC2 do not directly affect cardiomyocyte apoptosis or cardiac fibroblast activation, supporting an indirect role for ILC2 in post-MI cardiac repair. This study suggests the translational possibility to mitigate post-MI cardiac dysfunction by enhancing ILC2 growth, their Th2 cytokine production, and consequent eosinophil and dendritic cell accumulation in infarcted heart.
