# Supplementary material for: Group 2 innate lymphoid cells protect mouse heart from myocardial infarction injury via interleukin 5, eosinophils, and dendritic cells
Source: Cardiovasc Res. 2022 Sep 5;119(4):1046–61. doi: 10.1093/cvr/cvac144 (PMC10153644; doi:10.1093/cvr/cvac144)
Supplement: cvac144_Supplementary_Data [file cvac144_supplementary_data.pdf]

Supplemental Materials

Group 2 innate lymphoid cells protect mouse heart from myocardial infarction injury via interleukin 5, eosinophils, and dendritic cells

Tianxiao Liu,[1,2,3#] Zhaojie Meng,[2#] Jing Liu,[2,3#] Jie Li,[2] Yuanyuan Zhang,[2,4] Zhiyong Deng,[2] Songyuan Luo,[2] Minjie Wang,[2] Qin Huang,[2] Shuya Zhang,[2,4] Pauline Fendt,[2] Julie Devouassous,[2] Dazhu Li,[3] Andrew Neil James McKenzie,[5] Matthias Nahrendorf,[6] Peter Libby,[2] Junli Guo,[2,4] Guo-Ping Shi[2]

[1]Guangdong Provincial Geriatrics Institute, Guangdong Provincial People's Hospital, Guangdong Academy of Medical Sciences, Guangzhou 510080, China;
[2]Department of Medicine, Brigham and Women’s Hospital and Harvard Medical School, Boston, MA 02115, USA
[3]Laboratory of Cardiovascular Immunology, Institute of Cardiology, Union Hospital, Tongji Medical College, Huazhong University of Science and Technology, Wuhan 430022, China
[4]Institute of Cardiovascular Research, Key Laboratory of Emergency and Trauma of Ministry of Education, Hainan Medical University, Haikou 570100, China
[5]Medical Research Council Laboratory of Molecular Biology, Cambridge, UK
[6]Center for Systems Biology, Massachusetts General Hospital, Harvard Medical School, Boston, MA 02114, USA

#These authors contributed equally to this study.

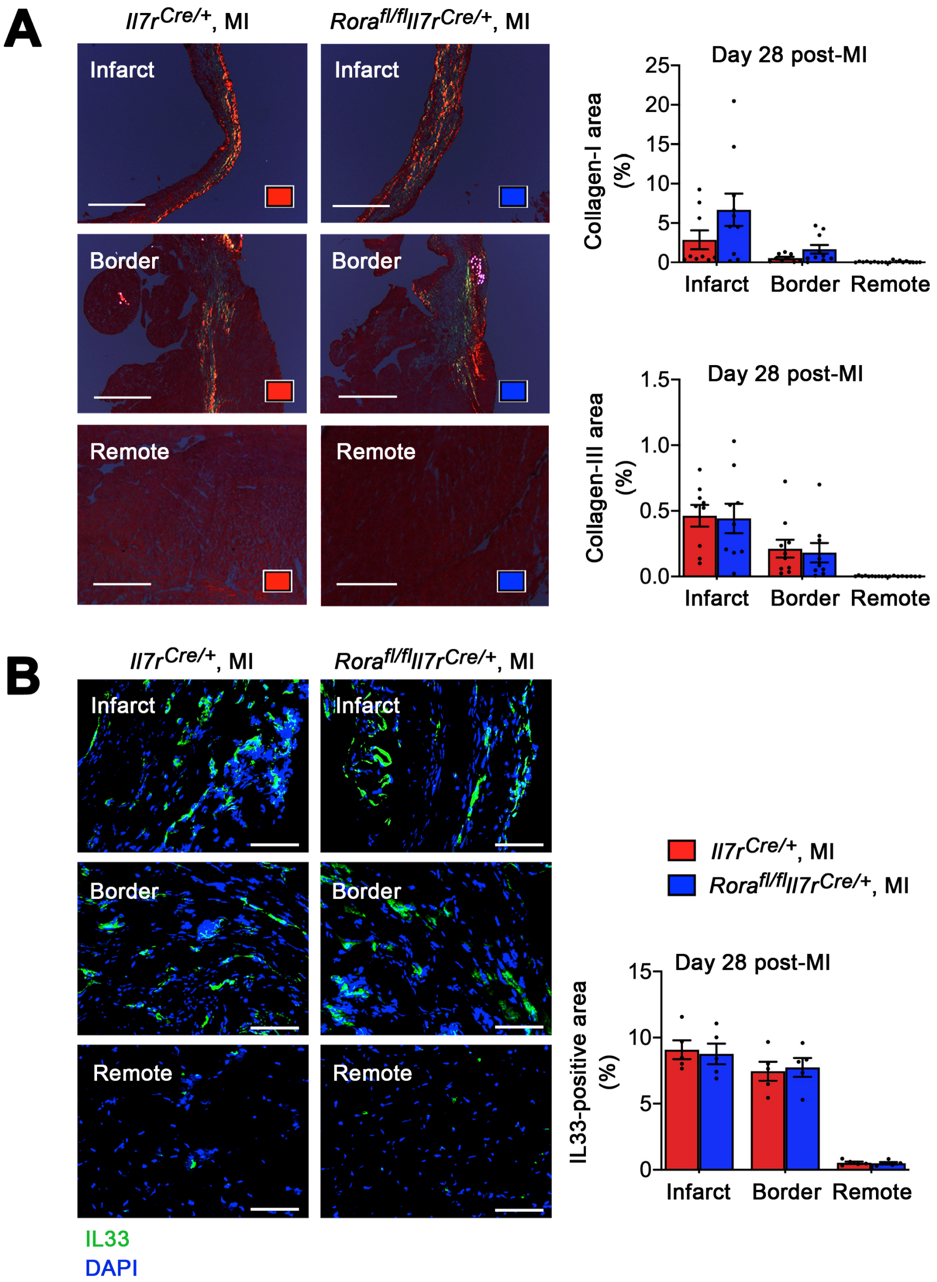

Supplemental Figure S1. Sirius red and immunofluorescent staining to detect collagen-I (red and orange) and collagen-III (green) (**A**) and IL33 expression (**B**) in the infarct, border, and remote regions of infarcted hearts from *Il7r*$^{Cre/+}$ and *Rora*$^{fl/fl}$*Il7r*$^{Cre/+}$ mice at 28 days post-MI. Scale: 200 µm in **A** and 50 µm in **B**. Data are mean±SEM. n=9~10 mice per group in **A** and n=5 mice per group in **B**, non-parametric Mann-Whitney *U* test followed by Bonferroni correction.

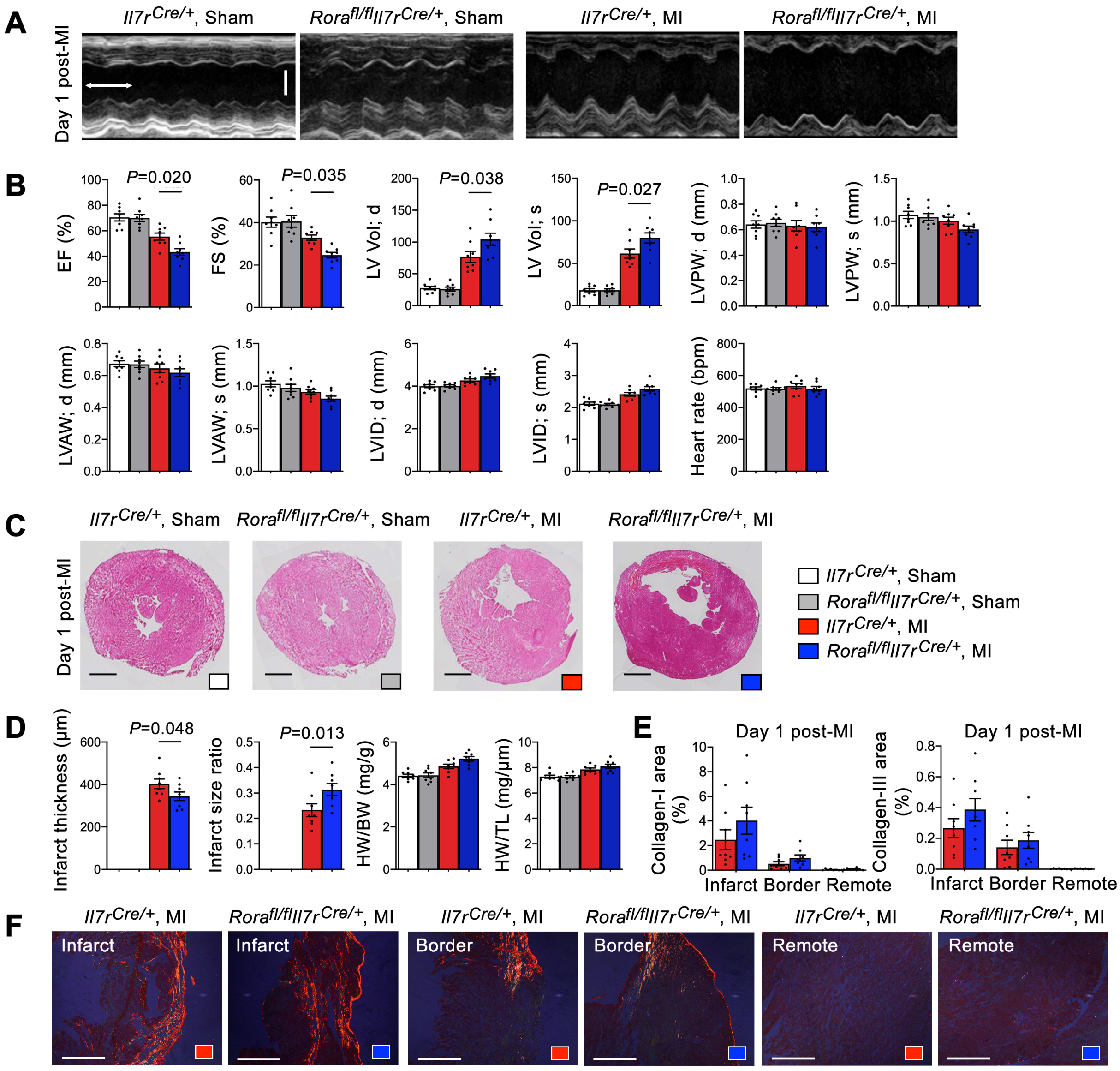

Supplemental Figure S2. ILC2 deficiency aggravates cardiac dysfunction at 1 day post-MI. **A.** Representative LV M-mode echocardiogram images of *Il7r*$^{Cre/+}$ and *Rora*$^{fl/fl}$*Il7r*$^{Cre/+}$ mice post-MI or sham as indicated (time stamp: 100 ms, scale: 1.7 mm). **B.** Cardiac functions post-MI or sham in different groups of mice. **C/D.** Infarct thickness, infarct size ratio and representative images (scale: 1.50 mm), BW/HW, and BW/TL post-MI or sham in different groups of mice. **E/F.** Sirius red staining was used to quantify collagen-I (red and orange) and collagen-III (green) in infarct, media, and remote regions from *Il7r*$^{Cre/+}$ and *Rora*$^{fl/fl}$*Il7r*$^{Cre/+}$ mice post-MI. Representative images are shown in **C** and **F** (scale: 200 µm). Data are mean±SEM. n=7~10 mice per sham group, n=15~20 mice per MI group, one-way ANOVA test followed by a post hoc Tukey's test (**B** and **D**), or non-parametric Mann-Whitney *U* test followed by Bonferroni correction (**E**).

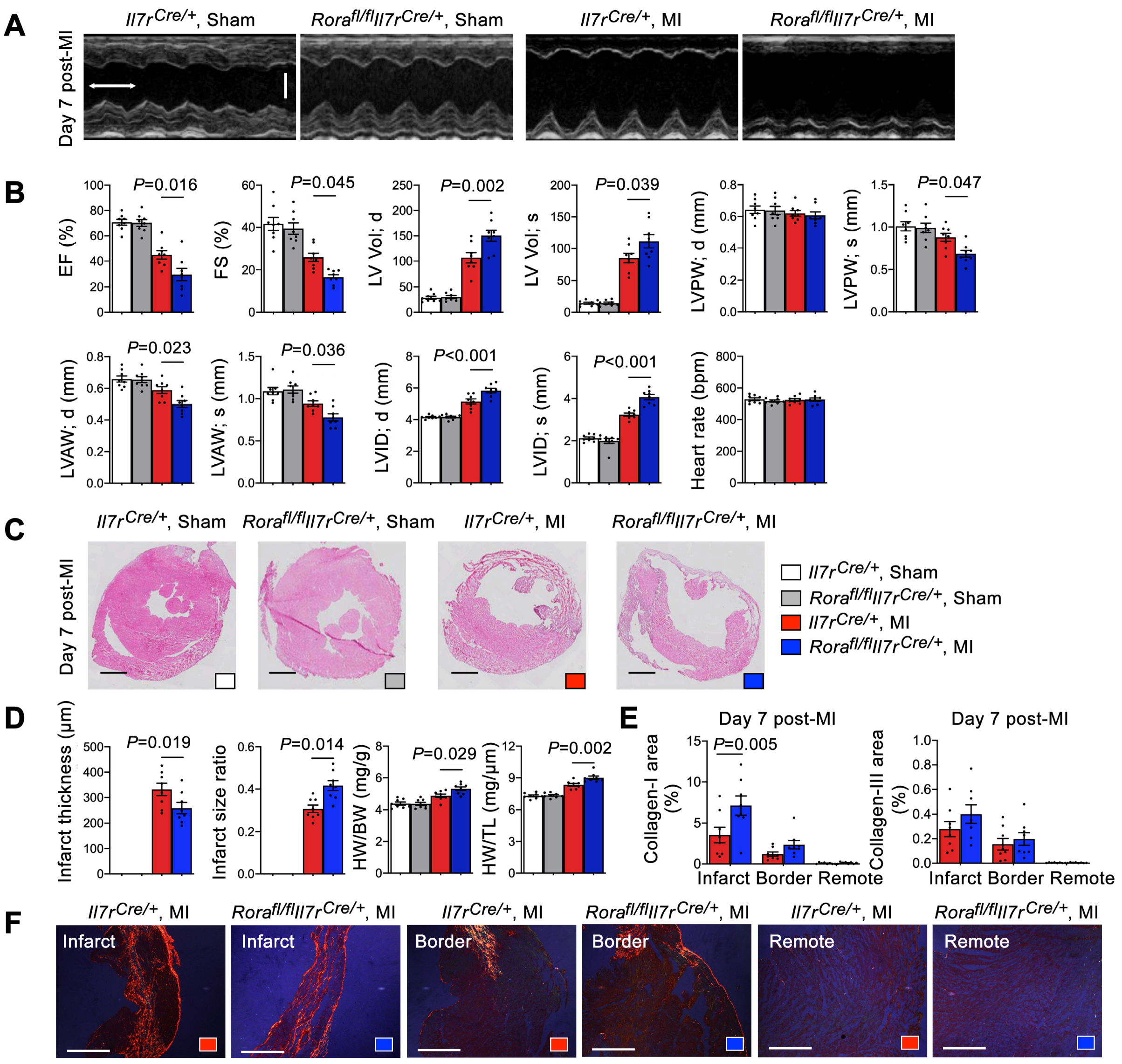

Supplemental Figure S3. ILC2 deficiency aggravates cardiac dysfunction at 7 days post-MI. **A.** Representative LV M-mode echocardiogram images of *Il7r*$^{Cre/+}$ and *Rora*$^{fl/fl}$*Il7r*$^{Cre/+}$ mice post-MI or sham as indicated (time stamp: 100 ms, scale: 1.7 mm). **B.** Cardiac functions post-MI or sham in different groups of mice. **C/D.** Infarct thickness, infarct size ratio and representative images (scale: 1.50 mm), BW/HW, and BW/TL post-MI or sham in different groups of mice. **E/F.** Sirius red staining was used to quantify collagen-I (red and orange) and collagen-III (green) in infarct, media, and remote regions from *Il7r*$^{Cre/+}$ and *Rora*$^{fl/fl}$*Il7r*$^{Cre/+}$ mice post-MI. Representative images are shown in **C** and **F** (scale: 200 μm). Data are mean±SEM. n=7~10 mice per sham group, n=15~20 mice per MI group, one-way ANOVA test followed by a post hoc Tukey's test (**B** and **D**), or non-parametric Mann-Whitney *U* test followed by Bonferroni correction (**E**).

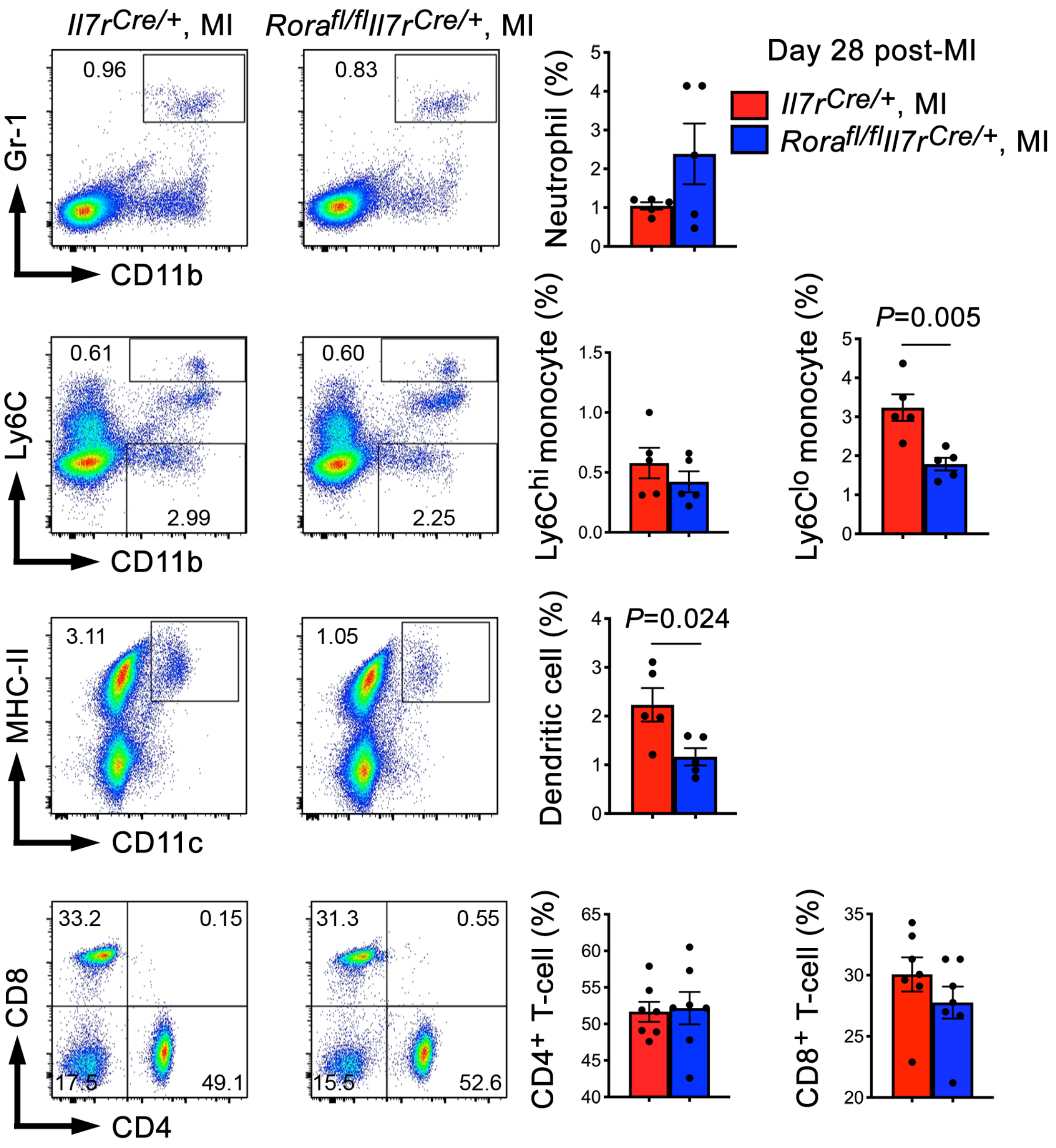

Supplemental Figure S4. FACS quantification of spleen immune cells in *Il7r*$^{Cre/+}$ and *Rora*$^{fl/fl}$*Il7r*$^{Cre/+}$ mice at 28 days post-MI. CD45^{+}CD11b^{+}Gr-1^{+} neutrophils, CD45^{+}CD11b^{+}Ly6C^{hi} monocytes, CD45^{+}CD11b^{+}Ly6C^{lo} monocytes, CD45^{+}CD11c^{+}MHC-II^{+} dendritic cells, CD45^{+}CD4^{+} and CD45^{+}CD8^{+} T cells from *Il7r*$^{Cre/+}$ and *Rora*$^{fl/fl}$*Il7r*$^{Cre/+}$ mice. Representative FACS images are shown to the left. Data are mean±SEM. n=5~7 mice per group, non-parametric Mann-Whitney *U* test followed by Bonferroni correction.

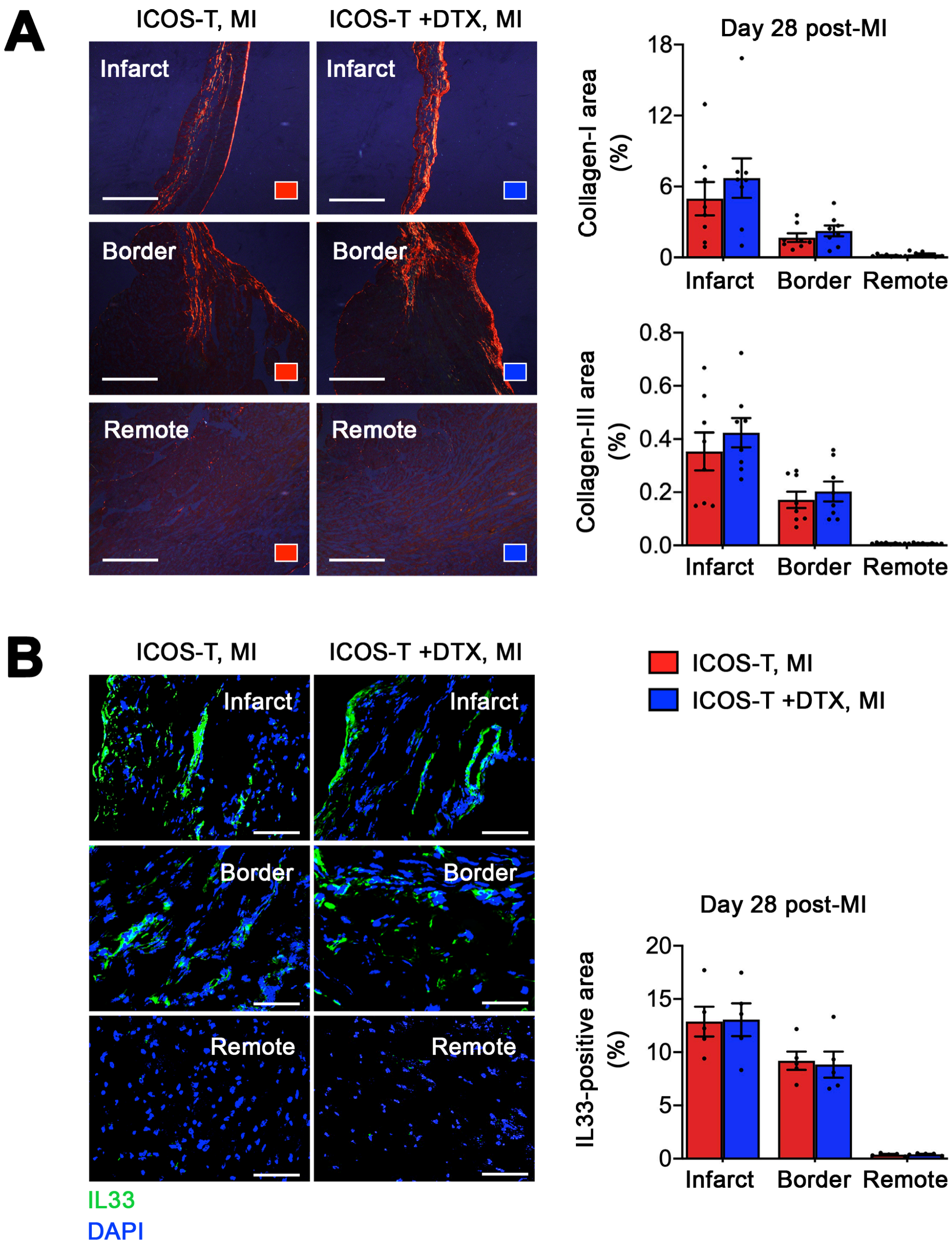

Supplemental Figure S5. Sirius red and immunofluorescent staining to detect collagen-I (red and orange) and collagen-III (green) (**A**) and IL33 expression (**B**) in the infarct, border, and remote regions of infarcted hearts from ICOS-T mice treated with or without DTX at 28 days post-MI. Scale: 200 µm in **A** and 50 µm in **B**. Data are mean±SEM. n=8 mice per group in **A** and n=5 mice per group in **B**, non-parametric Mann-Whitney *U* test followed by Bonferroni correction.

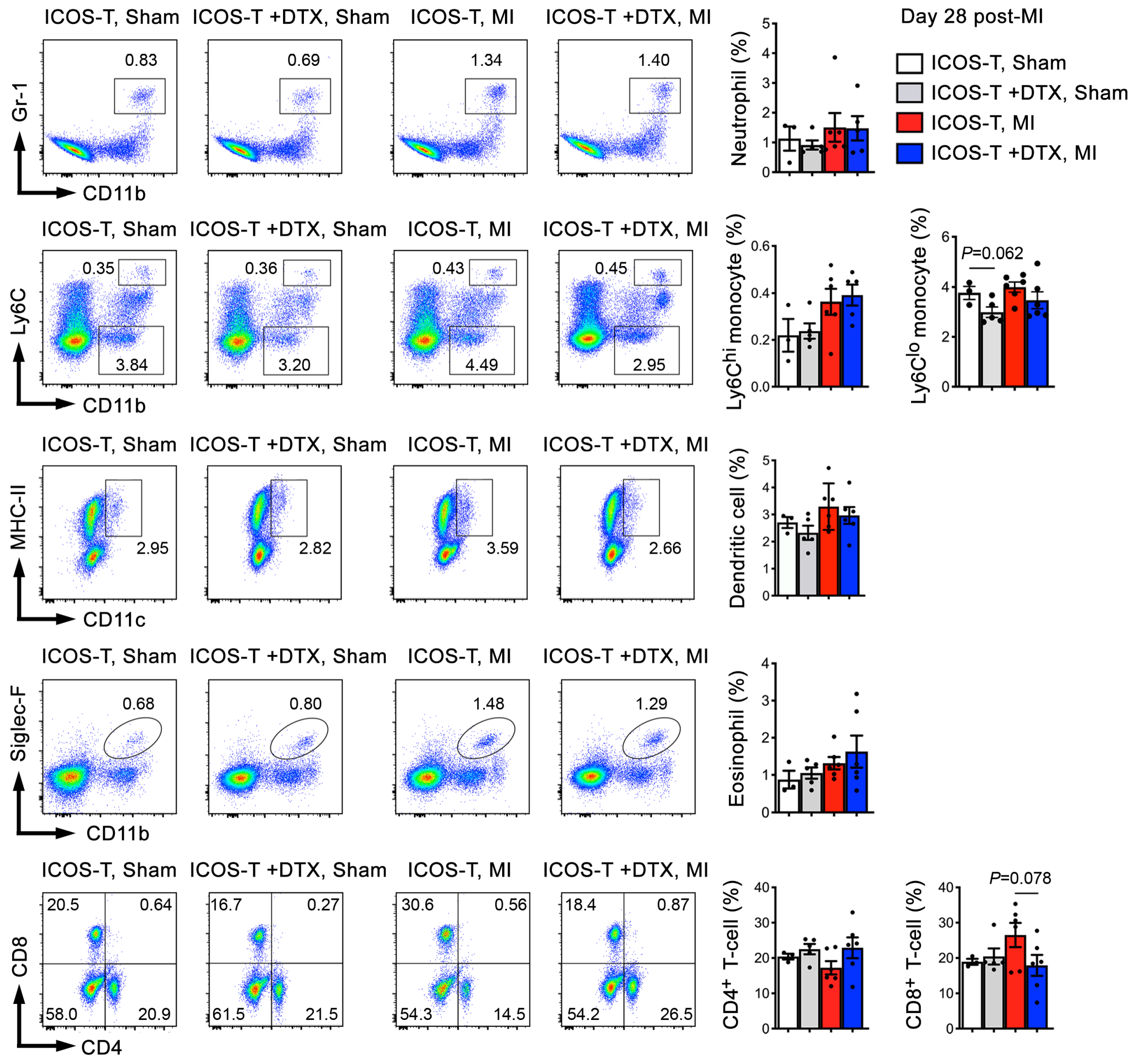

Supplemental Figure S6. FACS quantification of splenic immune cells in ICOS-T mice with different treatments as indicated at 28 days post-MI. $CD45^+CD11b^+Gr\text{-}1^+$ neutrophils, $CD45^+CD11b^+Ly6C^{hi}$ monocytes, $CD45^+CD11b^+Ly6C^{lo}$ monocytes, $CD45^+CD11c^+MHC\text{-}II^+$ dendritic cells, $CD45^+CD11b^+Siglec\text{-}F^+$ EOS, and $CD45^+CD4^+$ and $CD45^+CD8^+$ T cells. Representative FACS images are shown to the left. Data are mean±SEM. n=3~6 mice per group, one-way ANOVA test followed by a post hoc Tukey's test.

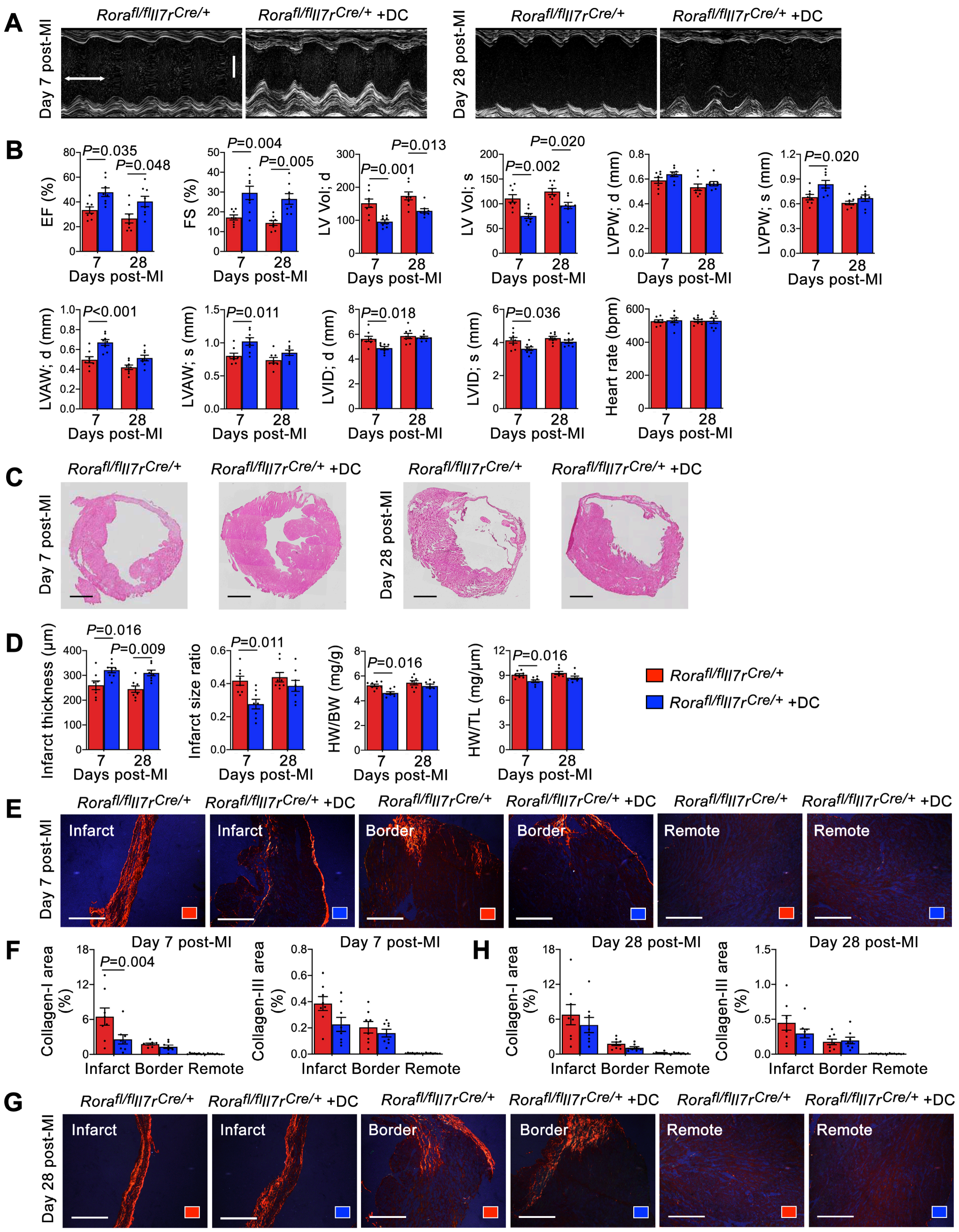

Supplemental Figure S7. Donor DC from WT mice protects cardiac functions in *Rora*$^{fl/fl}$*Il7r*$^{Cre/+}$ mice at 7 and 28 days post-MI. **A.** Representative LV M-mode echocardiogram images (time stamp: 100 ms, scale: 1.7 mm). **B.** Cardiac functions and heart rates. **C/D.** Infarct thickness, infarct size ratio, HW/BW, and HW/TL. Representative H&E staining images are shown in **C**. Scale: 1.50 mm. **E/F.** Sirius red staining of collagen-I (red and orange) and collagen-III (green) in infarct, media, and remote regions from *Rora*$^{fl/fl}$*Il7r*$^{Cre/+}$ mice at 7 days post-MI. **G/H.** Sirius red staining of collagen-I and collagen-III in infarct, media, and remote regions from *Rora*$^{fl/fl}$*Il7r*$^{Cre/+}$ mice at 28 days post-MI. Representative images are shown in **E** and **G** (scale: 200 μm). Data are mean±SEM. n=8 mice per group, non-parametric Mann-Whitney *U* test followed by Bonferroni correction.

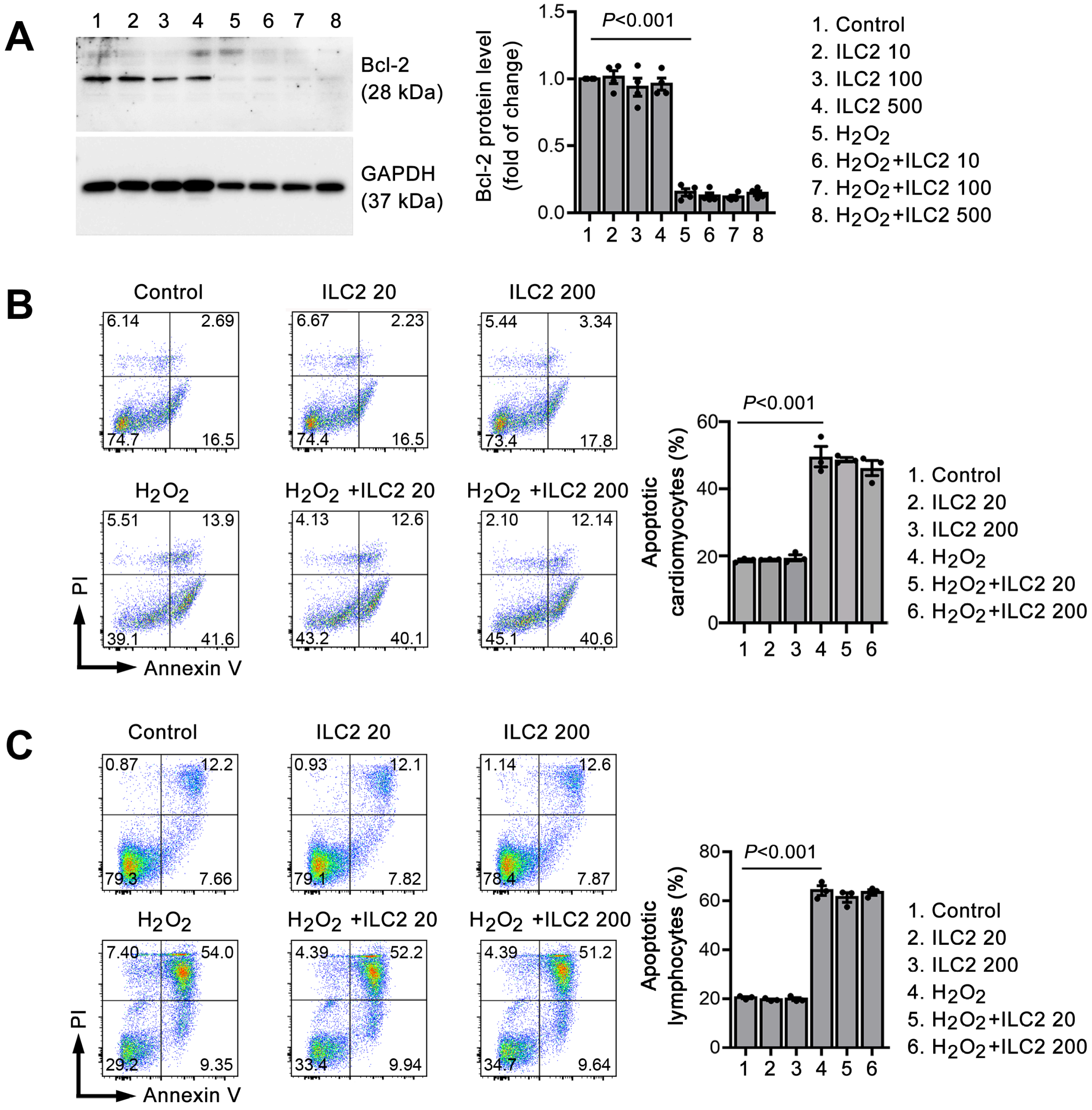

Supplemental Figure S8. ILC2 do not affect cardiomyocyte and lymphocyte death **A.** Immunoblot detected Bcl-2 expression in adult mouse cardiomyocytes treated with ILC2 lysates (equivalent to 0, 10^3, 10^4 and $5x10^4$ ILC2/ml) with or without H_2O_2 (100 μM) for 4 hrs. Representative immunblots are shown to the left. n=3-4 independent experiments. **B/C.** FACS analysis of mouse cardiomyocyte and lymphocyte apoptosis. Cardiomyocytes (**B**) and splenic lymphocytes (**C**) were treated with ILC2 lysates (equivalent to 0, $2x10^3$, $2x10^4$ ILC2/ml) with or without H_2O_2 (100 μM) for 4 hrs. Representative FACS images are shown on the left. n=3 independent experiments. Data are mean±SEM, one-way ANOVA test followed by a post hoc Tukey’s test.

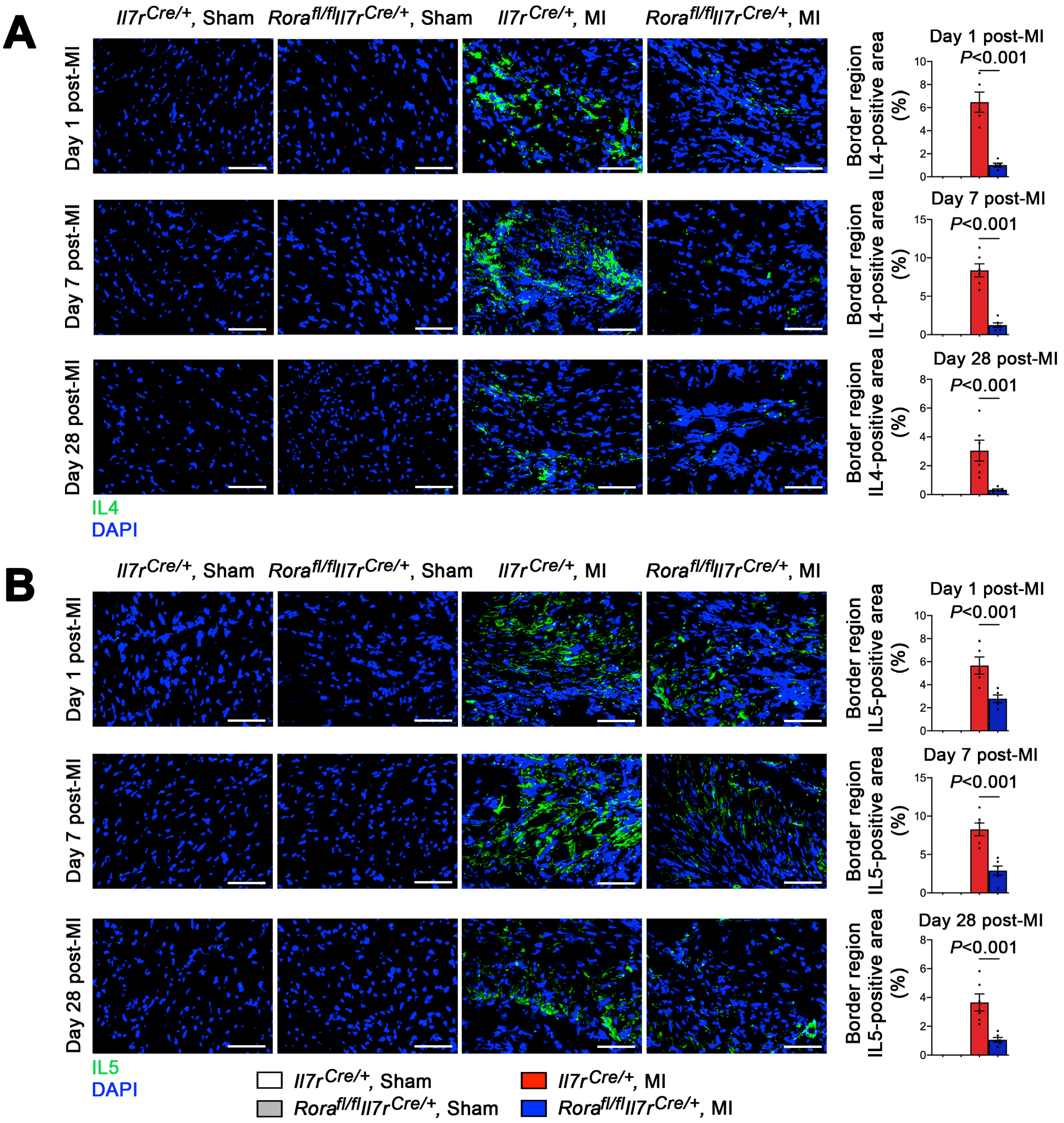

Supplemental Figure S9. Immunofluorescent staining, confocal imaging, and quantification of myocardial border region IL4 and IL5 levels in *Il7r*$^{Cre/+}$ and *Rora*$^{fl/fl}$*Il7r*$^{Cre/+}$ mice at 1, 7, and 28 days post-MI. **A.** IL4. **B.** IL5. Scale: 50 µm. Data are mean±SEM. n=5 mice per group, non-parametric Mann-Whitney *U* test followed by Bonferroni correction.

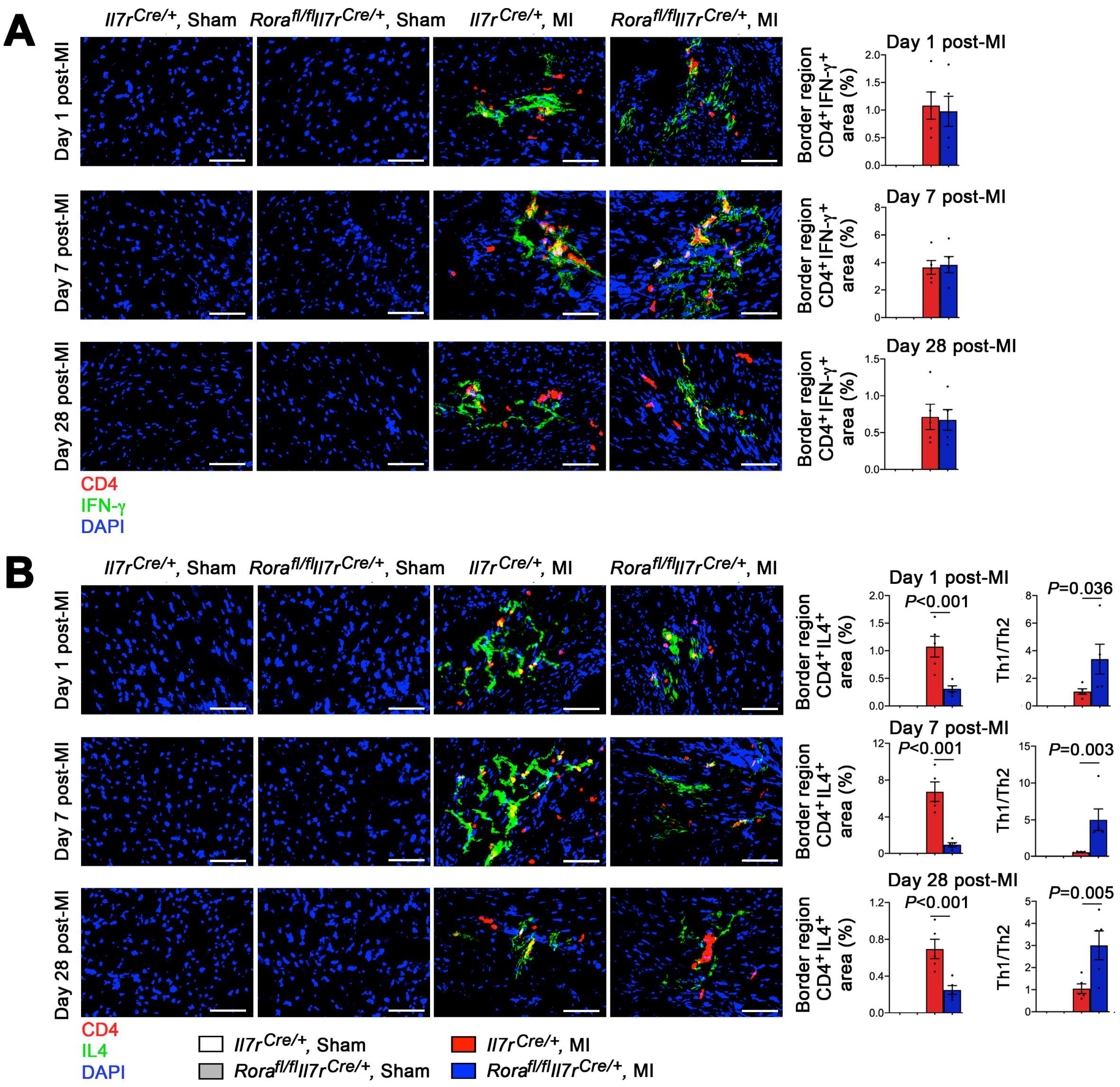

Supplemental Figure S10. Immunofluorescent double staining, confocal imaging, and quantification of myocardial border region Th1 and Th2 cells in *Il7r*$^{Cre/+}$ and *Rora*$^{fl/fl}$*Il7r*$^{Cre/+}$ mice at 1, 7, and 28 days post-MI. **A.** $CD4^+IFN\text{-}\gamma^+$ Th1 cells. **B.** $CD4^+IL4^+$ Th2 cells and Th1/Th2 ratio. Scale: 50 µm. Data are mean±SEM. n=5 mice per group, non-parametric Mann-Whitney *U* test followed by Bonferroni correction.

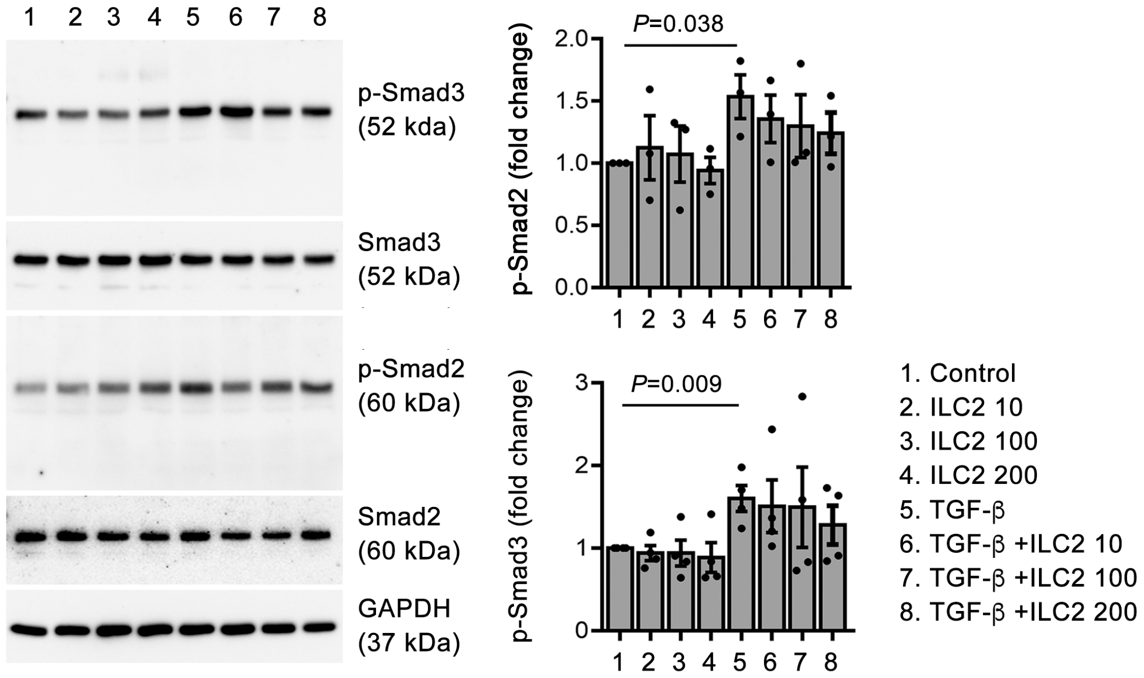

Supplemental Figure S11. ILC2 do not affect cardiac fibroblast Smad signaling. Immunoblots detected p-Smad2/3 and total Smad2/3 in mouse cardiac fibroblasts treated with ILC2 lysates (equivalent to 0, 10^3, 10^4 and $2x10^4$ ILC2/ml) with or without TGF-β (10 ng/ml) for 30 min. Representative immunoblots are shown to the left. n=5-7 independent experiments. Data are mean±SEM, one-way ANOVA test followed by a post hoc Tukey's test.
